# Closed-Loop Synergistic Nitric Oxide/Hydrogen Delivery with Feedback Control for Diabetic Wound Healing

**DOI:** 10.1007/s40820-026-02237-1

**Published:** 2026-05-26

**Authors:** Pengfei Wen, Pan Luo, Fuqiang Gao, Mingyi Yang, Junyou Li, Zhi Yang

**Affiliations:** 1 Department of Joint Surgery, Honghui Hospital, Xi’an Jiaotong University, Xi’an, 710054 People’s Republic of China; 2 Department of Plastic Surgery, Beijing Chaoyang Hospital Affiliated to Capital Medical University, Beijing, 100020 People’s Republic of China; 3 Center for Hip Preservation, Osteonecrosis and Developmental Dysplasia of the Hip, China-Japan Friendship Hospital, Beijing, 100029 People’s Republic of China; 4 Department of Orthopedics, China-Japan Friendship Hospital, Beijing, 100029 People’s Republic of China; 5 School of Mechanical Engineering, Sungkyunkwan University, Suwon, 16419 South Korea

**Keywords:** NO sensing, Anti-inflammatory hydrogen treatment, Closed-loop therapy, Decoupled-free coupling, Diabetic wound healing

## Abstract

Dynamic treatment represents the cornerstone of precision medicine. However, the development of closed-loop systems that respond to endogenous biomarkers remains challenging. Here, we report a dual-channel flexible electrocatalytic system (Pd-Ni_5_P_4_/DCEFS) integrated into a microneedle array that enables ultrasensitive real-time monitoring of nitric oxide (NO)—a key biomarker of wound inflammation—with a detection limit of 9.6 nM, while simultaneously driving the hydrogen evolution reaction with a low overpotential of − 91.0 mV at − 10 mA cm^−2^. This integrated platform establishes a closed-loop sensing-feedback-intervention mechanism, enabling NO-guided on-demand hydrogen generation for precision anti-inflammatory treatment. In diabetic mouse skin wound models, this adaptive hydrogen production strategy markedly suppresses inflammation, promotes tissue regeneration, achieves substantial wound closure within 5 days, and reduces the overall healing time to 11 days. This study establishes a new paradigm for closed-loop dynamic treatment of inflammatory diseases and provides a critical foundation for next-generation self-adaptive therapeutic platforms in regenerative medicine.

**Supplementary Information:**

The online version contains supplementary material available at 10.1007/s40820-026-02237-1.

## Highlights

- Pd-Ni_5_P_4_/dual-channel electrocatalytic flexible system (DCEFS) employs a bifunctional catalyst, dual-channel electrodes, and microneedle array to achieve continuous, decoupled-free coupling of nitric oxide sensing and hydrogen treatment.
- Pd-Ni_5_P_4_/DCEFS pioneers a single-cycle “sense-hydrogen treatment-sense” strategy for closed-loop inflammation resolution, delivering efficient reactive nitrogen species/reactive oxygen species clearance with real-time nitric oxide-based feedback for precise therapeutic control.
- Pd-Ni_5_P_4_/DCEFS innovatively establishes a closed-loop ‘sense-hydrogen treatment-sense’ paradigm guided by lesion-specific NO levels, enabling on-demand hydrogen intervention to potently resolve inflammation, robustly drive tissue regeneration, and markedly accelerate diabetic wound healing with excellent biocompatibility.

**Supplementary Information:**

The online version contains supplementary material available at 10.1007/s40820-026-02237-1.

## Graphical abstract

**Figure:**
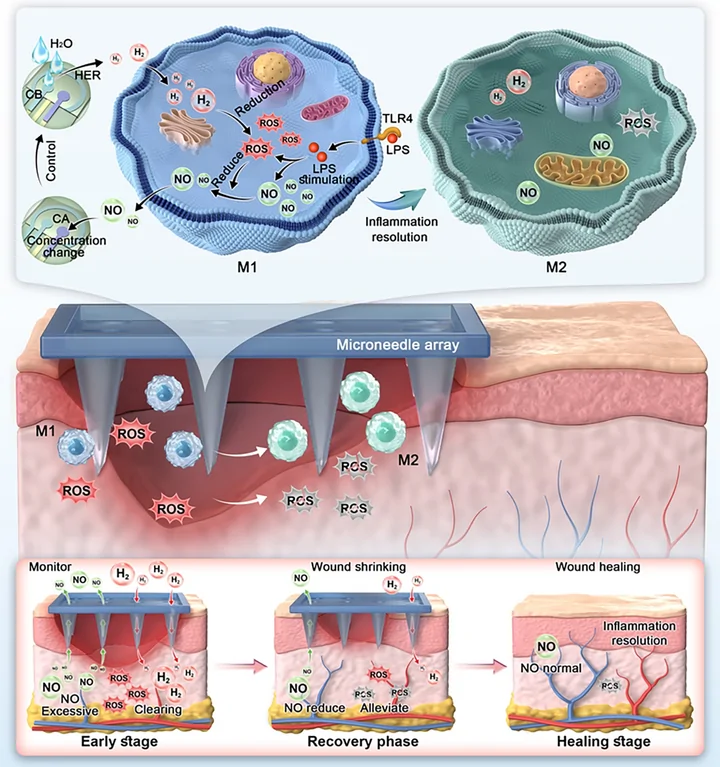

## Introduction

Precision medicine aims to deliver individualized and adaptive therapeutic strategies that optimize treatment efficacy and improve patient outcomes [1, 2]. Precision medicine for diabetes aims to tailor therapeutic strategies based on a patient's genetic background, lifestyle, and other unique characteristics, thereby enhancing clinical efficacy and minimizing complications. Recent research has further expanded the scope of this field: surface modification and functionalization of exosomes have introduced novel technical means for precision drug delivery [3]; innovative microfluidic technologies have provided robust platforms for the precise study and drug development of diabetic neuropathy [4]; and induced pluripotent stem cell (iPSC) models have demonstrated significant potential in advancing regenerative medicine research related to diabetes [5]. In this paradigm, closed-loop dynamic treatment systems that integrate real-time biomarker sensing with feedback-controlled intervention represent the cornerstone of next-generation medical technologies [6, 7]. These systems continuously monitor endogenous signals and dynamically modulate the release of therapeutic agents in response to evolving pathological states, thereby enabling precise control over conditions such as chronic inflammation [8–10]. Despite their conceptual promise, the development of fully integrated closed-loop platforms is hindered by the frequent mismatches between ultrasensitive sensing modules and high-performance therapeutic actuators. This decoupling typically results in compromised detection limits, delayed response times, sub-optimal treatment precision, and inadequate system-level coordination [11–15]. Consequently, seamless sensing–treatment integration is widely regarded as a critical requirement for translating closed-loop concepts into clinically viable precision therapies.

The impaired healing of diabetic wounds exemplifies a prototypical inflammation-driven pathology [16–21]. Within the wound microenvironment, nitric oxide (NO) serves as a key indicator of inflammatory status, rendering NO an ideal real-time biomarker for dynamic monitoring [22–28]. Concurrently, therapeutic hydrogen (H_2_) has emerged as a potent anti-inflammatory and proregenerative agent [29–33]. However, conventional hydrogen delivery modalities lack the capacity for real-time pathology-responsive regulation. Against this backdrop, diabetic wounds provide a compelling model for developing closed-loop systems in which the NO concentration acts as the input signal, and the rate of electrocatalytic hydrogen generation serves as the adaptive output [25–28, 34–36]. Such a platform would not only bridge the prevailing sensing–intervention divide in diabetic wound management but also establish a versatile technological framework extendable to other inflammation-associated disorders. Efficient and precise coordination between NO-monitored inflammatory dynamics and hydrogen-mediated therapeutic action hinges on the design of highly active and stable catalytic materials coupled with sophisticated system engineering [37, 38]. The successful implementation of this approach has substantial potential to transform the clinical management of refractory diabetic wounds and accelerate the broader adoption of personalized, closed-loop therapeutic strategies in precision medicine.

Herein, we developed a dual-channel flexible electrocatalytic platform integrated within a microneedle array for closed-loop management of diabetic wounds. The system incorporates a high-performance bifunctional Pd–Ni_5_P_4_ catalyst, electrolyte-supported microneedle substrate, and specialized dual-channel flexible electrode architecture fabricated via catalytic ink spin coating and gelatin-assisted bonding. This design enables two synergistic functions: (i) ultrasensitive, real-time electrochemical detection of NO-a pivotal inflammatory biomarker in the diabetic wound microenvironment-with a remarkable detection limit of 9.6 nM; and (ii) on-demand hydrogen evolution reaction (HER) for therapeutic H_2_ delivery, achieving a low overpotential of − 91.0 mV at − 10 mA cm^−2^. The closed-loop system directly employs the endogenous NO concentration as a feedback signal to dynamically modulate the rate of therapeutic hydrogen release, thereby establishing an integrated sensing–feedback–intervention loop. In a diabetic mouse model, NO-guided adaptive hydrogen treatment substantially attenuated inflammation within 5 days, accelerated wound closure despite reduced treatment frequency, and achieved complete re-epithelialization by day 11. These findings introduce a novel precision therapeutic paradigm for refractory diabetic wounds and provide a robust material and engineering framework for engineering self-adaptive closed-loop systems applicable to a broad spectrum of inflammatory and chronic diseases.

## Experimental Section

### Materials

Nickel(II) chloride hexahydrate (NiCl_2_·6H_2_O), ammonium fluoride (NH_4_F), urea (CH_4_N_2_O), sodium hypophosphite (NaH_2_PO_2_), sodium dihydrogen phosphate (NaH_2_PO_4_), disodium hydrogen phosphate (Na_2_HPO_4_), sodium nitrite (NaNO_2_), sodium hydroxide (NaOH), and sodium chloride (NaCl), palladium chloride (PdCl_2_), naphthol and potassium chloride (KCl) were purchased from Aladdin. Titanium foil (Ti foil, thickness = 0.1 mm) and gelatin were supplied by Sinopharm Chemical Reagent Co. Dual-channel electrocatalytic flexible electrode (DCEF, D = 2.0 cm), medical syringes and microneedle array are both purchased from supplier. Ti foil (thickness = 0.1 mm), Cu film, cupric sulfate (CuSO_4_·5H_2_O), hydrochloric acid (HCl), nitric acid (HNO_3_), alcohol (C_2_H_6_O), glucose (C_6_H_12_O_6_), ascorbic acid (AA), uric acid (UA), and hydrogen peroxide (H_2_O_2_) were supplied by Sinopharm Chemical Reagent. Glutaraldehyde (GA), L-arginine (L-Arg), L-NG-nitroarginine methyl ester hydrochloride (L-NAME), fetal bovine serum (FBS), and lipopolysaccharide (LPS) were purchased from Beyotime Biotechnology. Interleukin 4 (IL-4) and interferon-gamma (IFN-γ) were purchased from Sino Biological Inc. All of the chemicals were used as received without further purification.

### Preparation of Pd–Ni Precursor/Ti

To eliminate surface oxides, a 0.1 mm thick titanium foil was subjected to ultrasonic cleaning with concentrated HCl, deionized water, and C_2_H_5_OH. Subsequently, a solution was prepared by dissolving 0.9 mmol NiCl_2_, 0.1 mmol PdCl_2_, 2 mmol NH_4_F, and 5 mmol urea in 35.0 mL of water. The titanium foil (1.5 cm × 1.0 cm) was then immersed together with the prepared solution into a hydrothermal reactor, where the mixture was maintained at 85° C for 5.0 h. After cooling, the resulting Pd–Ni precursor/Ti composite was thoroughly rinsed with ethanol and water. Using the same method described above, replace 0.9 mmol NiCl_2_ and 0.1 mmol PdCl_2_ with 1.0 mmol NiCl_2_ to prepare the Ni precursor/Ti.

### Preparation of Pd-Ni_5_P_4_/Ti and Ni_5_P_4_/Ti

To prepare Pd-Ni_5_P_4_/Ti, the Pd–Ni precursor/Ti (Ni precursor/Ti) and 0.5 g of NaH_2_PO_2_ were placed in a ceramic boat, with NaH_2_PO_2_ positioned at the upstream end of the furnace. The sample was heated under a nitrogen atmosphere to 350.0 °C at a rate of 5 °C min^−1^ and held at this temperature for 3 h. Afterward, the furnace was cooled to room temperature (25 °C), and the sample was taken out. A Pd-Ni_5_P_4_/Ti (Ni_5_P_4_/Ti) composite was successfully obtained.

### Materials Characterizations

X-ray diffraction (XRD) patterns were obtained using a Rigaku Ultima IV diffractometer with Cu Kα radiation (λ = 1.54 Å) at a voltage of 40 kV and a current of 20 mA. High-resolution transmission electron microscopy (HRTEM) was performed on a JEOL JEM-2100F microscope operating at 200 kV. X-ray photoelectron spectroscopy (XPS) spectra were recorded using a Thermo Scientific ESCALAB 250Xi system with Al Kα radiation (1486.6 eV). Scanning electron microscopy (SEM) images were captured using a Hitachi SU8010 microscope.

### Transfer of Pd-Ni_5_P_4_/Ti to Pd-Ni_5_P_4_/DCEF

The treated Ti foil (m1) and the Pd-Ni_5_P_4_ catalyst grown on the substrate (Pd-Ni_5_P_4_/Ti, m2) were weighed to determine the mass of Pd-Ni_5_P_4_, calculated as m = m2—m1. A specified amount of Pd-Ni_5_P_4_ was scraped off the substrate for subsequent use. The Pd-Ni_5_P_4_ was then dispersed with 0.5 mg of carbon black in 1 mL of a water/ethanol mixture (volume ratio of 4:1), followed by the addition of 20.0 μL of Nafion solution (5.0 wt%). Carbon black was included to enhance the conductivity of the electrode. After ultrasonic treatment, the mass of DCEF (M1) was measured. A 5.0 μL portion of the dispersion was spin-coated (at 2500 rpm) onto the conductive surface of DCEF (1.0 × 1.5 cm^2^ area) until the mass of DCEF increased to M1 + m. The Pd-Ni_5_P_4_/DCEF electrodes were prepared.

### Integrated Dynamic Treatment System Pd-Ni_5_P_4_/DCEFS

After melting the gelatin, it is applied to the edges of the micro-needle array's electrolysis chamber. The gelatin-coated microneedle array is then swiftly attached to the prepared Pd-Ni_5_P_4_/DCEF. Once the gelatin has dried, the resulting structure is the Pd-Ni_5_P_4_/DCEFS. Subsequently, a small hole is created in the electrolysis chamber of the micro-needle array using the needle of a medical syringe to ensure a continuous supply of the electrolyte.

### Electrochemical Measurements

All electrochemical measurements were carried out on a CHI1040D Multichannel Electrochemical Analyzer (CH Instruments, Inc.) using a three-electrode configuration at room temperature. The dual-channel electrode consists of two working electrodes, along with a common reference electrode (Ag/AgCl) and a counter electrode (carbon). Linear sweep voltammetry (LSV) was conducted at a scan rate of 2 mV s^−1^ after the electrolyte was purged with O_2_ for 20 min. Electrochemical impedance spectroscopy (EIS) measurements were taken in the frequency range of 10^5^ Hz to 10^−2^ Hz. All potentials are referenced to the RHE using the Nernst equation: E(RHE) = E(SCE) + 0.0591 × pH + 0.224 V, unless specified otherwise. A 10.0 mM phosphate-buffered solution (PBS) was prepared by mixing 1.0 M K_2_HPO_4_ and 1.0 M KH_2_PO_4_ in a 2:1 volume ratio. Accelerated stability tests for HER were carried out in PBS over multiple cycles with a potential range from 0 to − 0.8 V (vs RHE) and a sweep rate of 100 mV s^−1^.

### Flexibility Test

In the flexibility test, the Pd-Ni_5_P_4_/DCEFS was wrapped around cylindrical surfaces with varying radii of curvature (ranging from 1.9 to 3.9 cm) and folded in half. Under preset parameters, bending was performed with a strain increment of 1.0 cm each time, while the relative change in resistance was measured using a multimeter.

### NO Solution Preparation and Calibration of Pd-Ni_5_P_4_/DCEF

*Gas Generation and Purification:* Following established methodologies [15, 39], NO gas was generated by the dropwise addition of 4 M H_2_SO_4_ into 2 M NaNO_2_ at a flow rate of 0.5 mL min^−1^. To ensure gas purity, the generated gas mixture was sequentially bubbled through 2 M and 4 M NaOH solutions to achieve the complete removal of NO_x_ impurities (primarily NO_2_).

*Solution Preparation:* Prior to gas saturation, the PBS buffer was purged with high-purity N_2_ for 30 min to remove dissolved oxygen. Subsequently, the purified NO gas was bubbled into the PBS at 20 °C for 30 min. Based on Henry’s Law and reported literature values [15, 39], the concentration of the resulting saturated NO stock solution was approximately 1.8 mM.

*Calibration and Validation:* A series of standard solutions were prepared via gradient dilution of the stock solution. Electrochemical calibration was performed at 0.75 V (vs. Ag/AgCl) using amperometry to measure oxidation currents, thereby establishing a linear relationship between current and concentration.

*Purity Assurance:* The potential interference from other nitrogen oxides was eliminated through multi-stage alkaline scrubbing and rigorous deoxygenation, ensuring the accuracy of the detection process.

### RAW 264.7 Sell Sulture

RAW 264.7 cells were cultured in a 5% CO_2_/95% air incubator (Series II 3111, Thermo Scientific, USA) in high-glucose Dulbecco’s modified Eagle medium (DMEM) supplemented with 10% fetal bovine serum (FBS), 1% penicillin, and 1% streptomycin at 37° C. For cell culture on Pd-Ni_5_P_4_/DCEFS, the Pd-Ni_5_P_4_/DCEFS was placed at the bottom of the cell culture solution, and cells were allowed to adhere to the surface of the material for 12 h. After 12 h, loosely bound RAW 264.7 cells were washed away. The electrodes with cells cultured for 12 h were then used for studies on NO release. For cell detection, RAW 264.7 cells were seeded onto Pd-Ni_5_P_4_/DCEFS at a density of 5.5 × 10^5^ cells cm^−2^.

### Cell Viability Assay

The cytotoxicity of Pd-Ni_5_P_4_ was evaluated using the CCK-8 assay. RAW 264.7 cells were seeded in a 96-well plate at 5000 cells per well and incubated for 24 h. After washing the cells three times with PBS, varying concentrations of Pd-Ni_5_P_4_ (Microneedle array, Pd-Ni_5_P_4_/DCEFS) were added, and the cells were cultured for another 24 h. Following this, 10 μL of CCK-8 reagent was added to each well, and the plate was incubated for about 4 h. Absorbance at 450 nm was measured using a microplate reader (Tecan, Austria).

### Real-Time Monitoring of NO Release During Cell

The Pd-Ni_5_P_4_/DCEFS sensor was co-cultured with RAW 264.7 cells. Both L-Arg and L-NAME (NO synthase inhibitor) were added at concentrations of 10.0 mM. The electrode potential was maintained at + 0.75 V vs. Ag/AgCl for amperometric detection of cell responses, as this potential corresponded to the plateau current for NO oxidation on the Pd-Ni_5_P_4_/DCEF electrodes.

### RAW 264.7 Cells Internalization

RAW 264.7 cells were seeded onto Pd-Ni_5_P_4_/DCEFS at a density of 5.5 × 10^5^ cells cm^−2^ in 35 mm diameter cell culture dishes. The cells were then polarized to either an antitumorigenic phenotype (M1) by treatment with 100 ng mL^−1^ LPS and 20 ng mL^−1^ IFN-gamma for 24 h, or to a pro-tumorigenic phenotype (M2) by treatment with 20 ng mL^−1^ mouse recombinant IL-4 for 24 h.

### Measurement of Faraday Efficiency

The faradaic efficiency was determined using simultaneous GC measurements (SP7800, China) and calculated by comparing the amount of H_2_ generated during potentiostatic cathodic electrolysis with the theoretical amount of H_2_, assuming 100% faradaic efficiency.

### Operational Raman Spectroscopy Measurements

Raman spectra were recorded using a LabRAM HR Evolution (HORIBA Scientific) spectrometer. The electrochemical cell for Raman measurements was custom-made from Teflon, with a quartz plate used as the laser window. A carbon electrode and an Ag/AgCl electrode (with 1.0 M KCl as the inner filling electrolyte) served as the counter electrode and reference electrode, respectively. To apply a controlled potential to the catalyst during Raman measurement, chronoamperometry was performed at various potentials.

### Research on H_2_ Treatment

After sterilization, the three-electrode system (working electrode: Pd-Ni_5_P_4_/DCEFS, B channel, reference electrode: 3.8 mm) and the associated electrochemical workstation equipment were positioned on the cell culture platform. The appropriate cell culture medium was then added to the culture dish, and hydrogen gas was continuously generated using the i-t (current–time) method. Once the current curve stabilized, cells that had been incubated for 12 h were transferred to fresh culture medium for hydrogen treatment under various conditions (e.g., voltage, time, LPS, SIN-1).

### DCFH-DA Probe for Detecting Reactive Oxygen Species in H_2_-Mediated Anti-inflammatory Effects

We employed 2',7'-dichlorofluorescin diacetate (DCFH-DA) as a green fluorescent probe to monitor ·OH levels, using Fenton's reagent to initiate intracellular ·OH production. The fluorescence signal in the cells corresponds to the green fluorescence generated when DCFH-DA reacts with reactive oxygen species (ROS) and other reactive substances. Upon entering the cells, DCFH-DA is deacetylated by intracellular esterases into a non-fluorescent compound, which is then oxidized by ROS/reactive nitrogen species (RNS) into 2',7'-dichlorofluorescein. This fluorescence signal was primarily used to detect changes in the levels of reactive species, allowing us to investigate the role of H_2_ in the anti-inflammatory process, particularly its ability to simultaneously neutralize relevant reactive species.

### Fluorescence Imaging and Quantitative Analysis

Fluorescence imaging was employed to quantify ROS levels. It flows a bit better now. To ensure the reliability of the quantification, regions with uniform cell density (approximately 100 cells per field) were selected for imaging. The fluorescence intensity was quantified by calculating the average intensity per cell using the software provided with the imaging instruments. All imaging parameters were kept constant during the calibration and data collection process to ensure comparability. Each experiment was performed in at least three independent replicates to minimize potential accidental or false-positive signals.

### Statistical Analysis

Two-way analysis of variance (ANOVA) was utilized to evaluate the significance of differences across various treatment groups under different experimental conditions or time points (e.g., stages, days, and types of induction agents). The ANOVA method was applied to analyze intra-group and inter-group variances, determining whether the effects of individual factors and their interactions on the results were statistically significant. Following the identification of significant differences via ANOVA, Tukey’s or Sidak’s post-hoc tests were further performed for pairwise comparisons of group means. Specifically, for the wound healing assays, the interaction between time and treatment groups was prioritized; for the reactive species scavenging experiments, differences between the treatment and control groups under various induction conditions were compared. All data are presented as mean ± standard deviation (SD), with sample sizes derived from four independent replicates. Statistical significance is denoted as follows: *p < 0.05, **p < 0.01, ***p < 0.001, and ns for non-significant differences. All statistical analyses were conducted using GraphPad Prism software.

### Detection and Quantification of Hydroxyl Radicals (⋅OH) Using DCFH-DA and Fenton's Reagent

We have used Fenton’s reagent to induce the generation of hydroxyl radicals (·OH) within cells, while continuous supply of hydrogen can efficiently scavenge ·OH and significantly reduce the intensity of green fluorescence signals. We used 2′,7′-dichlorodihydrofluorescein diacetate (DCFH-DA) as a green fluorescence probe to detect the levels of ·OH, with Fenton’s reagent as the inducer of ·OH generation inside cells. We processed the fluorescence signals in the images using the software provided by the instrument to obtain the average fluorescence intensity.

### Dynamic Therapeutic Effects of the A Channel (NO sensor) of Pd-Ni_5_P_4_/DCEFS at the Cellular Level

After co-culturing Pd-Ni_5_P_4_/DCEF electrodes (A channel) with RAW 264.7 cells for 12 h, current signals were first collected. To investigate the impact of different treatments on the cellular current response, the cells were divided into three groups: Group 1: LPS and IFN-γ was added to the culture medium to induce differentiation of the cells into M1 macrophages, and the changes in current signals were recorded. Group 2: IL-4 was added to the culture medium to induce differentiation of the cells into M2 macrophages, and the current signals were monitored. Group 3: This was the control group, where cells were left untreated. After incubation, current changes were observed. By comparing the current signal changes across these three groups, the electro-physiological response of the cells to different stimuli was evaluated. Additionally, fluorescence imaging was performed to assess the degree of cell differentiation in each group.

### A Single-Cycle Dynamic Treatment of Pd-Ni_5_P_4_/DCEFS at the Cellular Level

**Part 1: Multichannel Electrochemical Analyzer Setup**

First, open the Multichannel Electrochemical Analyzer control panel and select the dual-channel program settings.

1. Channel A Settings: Set to nitric oxide sensing mode, with a potential of 0.75 V and a duration of 400 s.
2. Channel B Settings: Next, proceed to the second channel and set it to hydrogen evolution mode, with a potential of − 0.25 V and a duration of 35 min.

This setup ensures a seamless transition between the nitric oxide sensing and hydrogen evolution programs.

**Part 2: System Setup**

Clamp the electrodes corresponding to the A and B channels of the Pd-Ni_5_P_4_/DCEF adapter onto the working electrode simultaneously.

1. Initial Setup: Place the system in co-culture with RAW 264.7 cells for 12 h. During this period, use channel A to collect the current signal.
2. LPS Addition: After 12 h, add LPS to the cell culture medium and use channel A to record the changes in the current signal.
3. End of Signal Collection: Once the current signal collection is complete, proceed to the hydrogen evolution setup.
4. Hydrogen Evolution: Use channel B to release hydrogen gas and perform hydrogen evolution.
5. Signal Collection Recovery: After hydrogen evolution, wait for 20 min, then use channel A to collect the current signal again, completing one cycle of testing.

This process ensures the system can successfully complete one full cycle of testing.

### Diabetic Mouse Model Building and Treatment

Streptozotocin (STZ) was dissolved in a citric acid and sodium citrate buffer for the induction of diabetes in mice. Mice were administered intraperitoneal injections of STZ (180 mg kg^−1^) daily until their blood glucose levels remained consistently above 20 mM after 14 days. Blood glucose levels were measured following an overnight fast. For the diabetic mice, circular wounds (0.5 cm in diameter) were created on the depilated dorsal skin to facilitate subsequent treatment.

### Multi-Layered Fixation Strategy

Interfacial Self-Adhesion: The inclusion of gelatin endows the microneedle patch with excellent tissue-adhesive properties, providing the primary layer of stable mechanical anchoring between the electrode and the wound bed.

Dual External Reinforcement: Following patch implantation, medical-grade breathable tape was applied over the patch area to prevent initial displacement. Subsequently, the animal's torso was wrapped with a medical elastic bandage. This setup not only provides consistent downward pressure to ensure continuous microneedle penetration but also serves as a physical barrier against scratching.

Adapter Strain-Relief Fixation: To mitigate mechanical stress on the interface, the sensor's signal adapter was securely fastened to the animal's dorsal skin (primarily in the interscapular region) using medical tape. By transferring the hardware weight to the animal's torso rather than the electrode interface, we effectively eliminated contact instability caused by wire tension.

Control of Environmental Interference: During the experimental period, all animals were housed individually in separate cages. This measure successfully prevented equipment damage resulting from social interactions, such as biting or play among cage mates, thereby ensuring the continuity and integrity of signal acquisition.

### Dynamic Therapeutic Effects of Pd-Ni_5_P_4_/DCEFS at the in vivo Level

The mice were randomly divided into two groups (*n* = 4). The treatment protocols for the experimental and control groups are as follows:

**Experimental Group:**

The Pd-Ni_5_P_4_/DCEF electrode was used for anti-inflammatory treatment of diabetic mice wounds under optimized voltage and time conditions. Initially, the A channel of the Pd-Ni_5_P_4_/DCEF electrode was used to monitor current changes due to the variation in nitric oxide concentration at the wound site of the diabetic mouse. Subsequently, the B channel was activated to release hydrogen gas for 35 min. After the hydrogen release phase, a 20-min waiting period was observed before the A channel was used again to monitor the current changes induced by nitric oxide at the wound site. Based on the current fluctuations, the clearance of wound inflammation was assessed. This process constitutes one complete cycle, with multiple treatments performed on diabetic mice daily.

**Control Group:**

First, the A channel of the Pd-Ni_5_P_4_/DCEF electrode was used to monitor the current changes caused by variations in nitric oxide concentration at the wound site of the diabetic mice. The Pd-Ni_5_P_4_/DCEF electrode was then placed at the wound site without hydrogen gas release. After a 20 min waiting period, the A channel was used again to monitor the current changes induced by nitric oxide at the wound site, and the clearance of wound inflammation was assessed based on the current fluctuations. This experimental design aims to compare the differences in wound inflammation clearance between the treatment and control groups, thereby evaluating the anti-inflammatory effects of the Pd-Ni_5_P_4_/DCEF electrode.

### Algorithmic Automation

**System Sterilization and Application**

Sterilization: To ensure an aseptic experimental environment, the external adapter and electrode components of the Pd-Ni_5_P_4_/DCEFS were disinfected with medical-grade alcohol, followed by ultraviolet (UV) irradiation sterilization and subsequent drying.

**Semi-Automated Feedback Treatment Protocol**

Automated Execution: The predefined automated treatment program was initiated, transitioning the system into a synchronized mode for Channels A and B. In this phase, nitric oxide current signals were automatically collected (monitoring) while concurrently triggering electrocatalytic hydrogen release (therapy). This stage required no manual intervention, ensuring instantaneous therapeutic response. Annual Evaluation and Feedback: This feedback mechanism followed the logic of "algorithmic collection and manual decision-making." Operators extracted the NO signal curves recorded by Channel A in real-time and, in conjunction with the macroscopic observation of wound healing, evaluated the extent of inflammatory clearance. Parameter Optimization and Secondary Cycling: A 20-min resting period was established prior to the initiation of the second cycle. This phase served a dual function: first, it provided sufficient duration for the electrochemically generated hydrogen to scavenge inflammatory mediators; second, it allowed the electrode interface to re-equilibrate, ensuring the baseline stability and accuracy of subsequent NO current signal acquisition. Following this 20-min stabilization period, operators manually fine-tuned the electrochemical parameters (such as potential amplitude or pulse duration) based on the inflammatory clearance time and assessment results from the initial round, subsequently initiating the second therapeutic cycle. Closed-Loop Healing Assessment: By comparing the evolution of NO signals across the two cycles, the progression of wound healing was systematically evaluated, thereby completing a comprehensive closed-loop treatment cycle.

## Results and Discussion

### Design Principle and Structural Characterizations

Figure S1 shows the Pd-doped Ni_5_P_4_ catalyst (Pd-Ni_5_P_4_) synthesized by phosphidation of a Pd-doped nickel-based precursor. The detailed synthesis methodology is presented in the Experimental section. X-ray diffraction (XRD) was used to analyze the crystallographic characteristics of the samples. Notably, diffraction peaks corresponding to the Ti foil (PDF#65–3362) arose at angles of 35.1°, 38.4°, 40.2°, and 53.0° (Figs. S2 and S3). The predominant diffraction peaks at 15.2°, 30.4°, 34.5°, 36.1°, 44.2°, 45.2°, 47.1°, 48.0°, and 54.0° correspond to the (100), (200), (202), (104), (212), (204), (301), (213), and (220) crystal planes of orthorhombic Ni_5_P_4_ (PDF#18–0883). Compared with the reference values, the incorporation of Pd was associated with slight shifts in the (100) and (212) peaks. This observation aligns with previous findings [40], indicating that Pd incorporation influences the lattice structure of Ni_5_P_4_ (Fig. S4). XPS analysis of the Pd-Ni_5_P_4_ catalyst (Fig. S5 and Table S1) reveals characteristic peaks for Ni, Pd, and M-P bonds, confirming the successful integration of Pd into the phosphide matrix and demonstrating a strong electronic coupling effect that is expected to optimize the active sites for enhanced bifunctional catalytic performance.

Scanning electron microscopy (SEM) analysis revealed that the microstructure of the palladium-doped Ni_5_P_4_ catalyst supported on titanium (Pd-Ni_5_P_4_/Ti) exhibits a distinctive flower-like morphology composed of nanosheets, as shown in Figs. S6-S9. Each flower-like microsphere measures ~ 10 μm in diameter. Transmission electron microscopy (TEM) further revealed that the Pd-Ni_5_P_4_ structure primarily adopts a sheet-like configuration. High-resolution TEM images indicate a lattice spacing of ~ 0.56 nm, corresponding to the (002) crystal plane of Ni_5_P_4_, as shown in Fig. S8. Moreover, scanning TEM combined with energy-dispersive X-ray spectroscopy (STEM-EDS) confirmed the successful synthesis of Pd-Ni_5_P_4_ and reveals a homogeneous distribution of palladium (Pd), nickel (Ni), and phosphorus (P) within the microspheres, as illustrated in Fig. S9. These results demonstrate successful Pd doping and its impact on the Ni_5_P_4_ structure.

### Synthesis of Pd-Ni_5_P_4_/DCEF via Catalytic Ink Spin-Coating Method

**Fig. 1:**
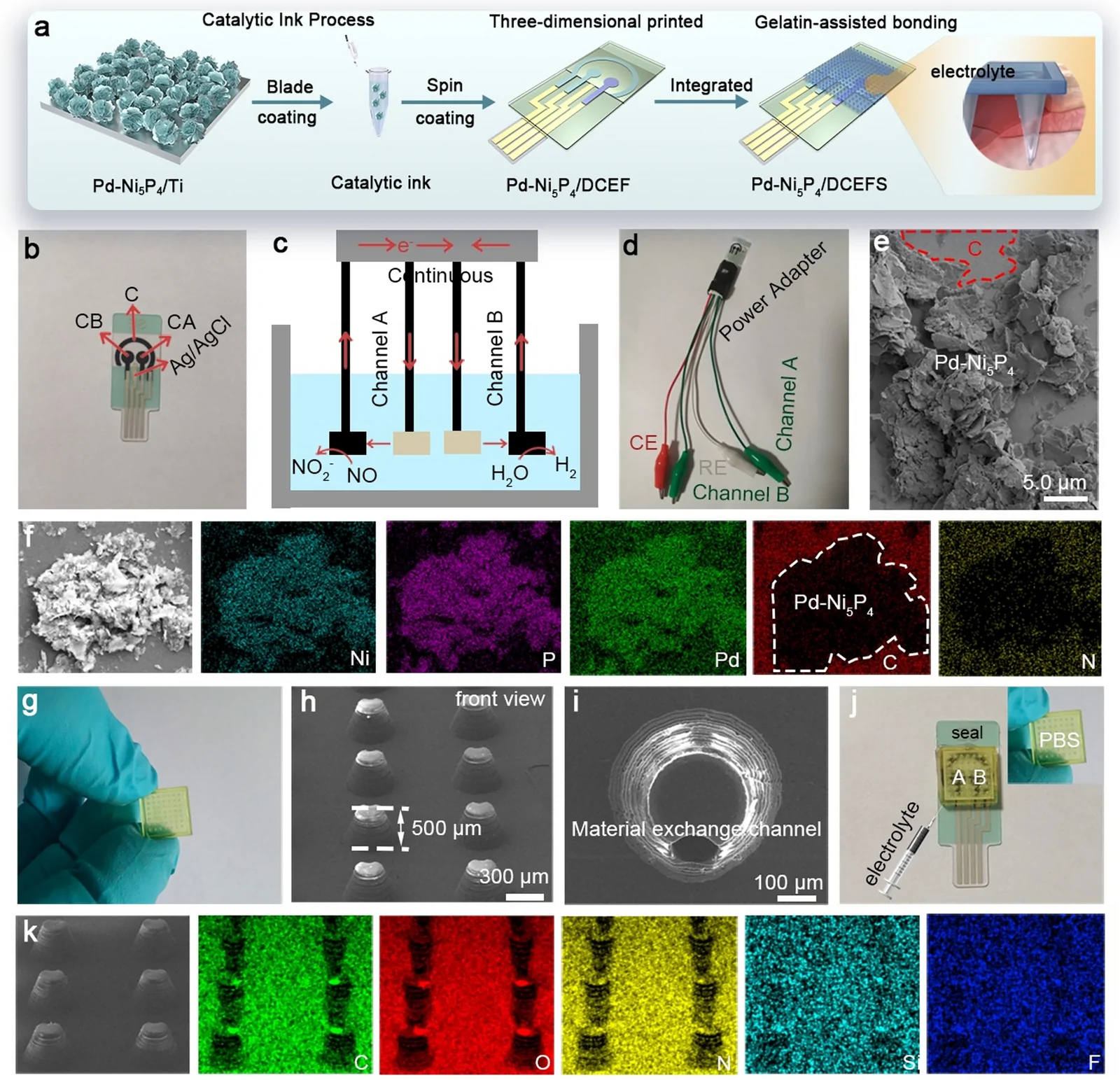
Schematic illustration of the construction and structural characterization of the Pd-Ni_5_P_4_/DCEFS integrated system for dynamic monitoring/therapy. **a** Preparation of the dual-channel system. **b** Physical image of DCEF. **c** Schematic of the DCEF working principle. **d** Physical image of DCEF integrated with an adapter. **e** SEM image of Pd-Ni_5_P_4_ transferred onto DCEF using the catalytic ink process. **f** SEM-mapping image of Pd-Ni_5_P_4_/DCEF. **g** Physical image of the microneedle array. **h** SEM image of pores in the microneedle array. **i** SEM image of a single pore in a microneedle array. **j** Physical image of Pd-Ni_5_P_4_/DCEFS, with the inset showing the PBS solution in the microneedle array. **k** SEM-mapping image of microneedle array

The Pd-Ni_5_P_4_/Ti electrode has a rigid substrate in the form of a titanium foil, which limits its applicability in studies related to biological systems. To address this limitation, we used a three-dimensional printed dual-channel electrocatalytic flexible electrode (DCEF) as the substrate (Fig. 1a, b). This novel electrode demonstrates exceptional flexibility, allowing it to undergo substantial bending deformation, fulfilling the requirements for skin conformation in biological applications (Fig. S10). The DCEF was engineered to have two distinct working electrodes, denoted as electrodes A and B, which serve the respective functions of channels A and B (Fig. 1b, c). This design not only enhances the electrode's compatibility with biological environments but also facilitates versatile operational capabilities. With the development of program settings for electrocatalysis instruments, continuous dual-channel programming is now achievable, enabling sustained and controllable electrocatalytic reactions in complex channels, such as dual-channel systems. Specifically, the A channel was used for real-time NO monitoring, whereas the B channel was employed for the continuous and controllable evolution of hydrogen (Fig. 1b). The electrode design enabled continuous NO detection and hydrogen evolution, providing instrumental support for the cyclic operation of dynamic treatment systems. These two channels of Pd-Ni_5_P_4_/DCEF can be set individually or simultaneously and integrated via a multifunctional adapter (Fig. 1c). When the A- and B-channels operate simultaneously, the corresponding working electrode clamp of the adapter must be attached to the working electrode. However, when either the A or B channel operates independently, only a single-electrode clamp is required on the working electrode (Fig. 1d). This design facilitates the integration of dual-channel electrocatalytic flexible electrodes with continuous workstation programming.

During the preparation process of Pd-Ni_5_P_4_/DCEF, we employed a catalytic ink method to successfully transfer the Pd-Ni_5_P_4_ catalyst from the Ti foil to the DCEF electrode (Fig. 1a). Through prolonged ultrasonic treatment, the Pd-Ni_5_P_4_ catalyst morphology transformed from micron-sized spherical to irregular nanosheet-like structures, as confirmed by the SEM images (Fig. 1e). Despite this morphology change, its performance remained essentially the same, in agreement with a previous study [41]. To confirm the successful transfer of Pd-Ni_5_P_4_ to the DCEF electrode, we conducted SEM elemental mapping of Pd-Ni_5_P_4_/DCEF. The results show the distributions of Pd, Ni, and P. Notably, carbon (C) from the carbon-based DCEF electrode was distinctly distributed around the Pd-Ni_5_P_4_ material, evidencing the transfer of Pd-Ni_5_P_4_ (Fig. 1f).

### Integration of Pd-Ni_5_P_4_/DCEFS Using Gelatin

To construct a portable substance-exchange platform, we designed a system based on a microneedle array for hydrogen release and NO exchange. The microneedle array had dimensions of 1.0 × 1.0 cm^2^ (Fig. S11), and the base was equipped with an electrolyte reservoir that could store a certain amount of electrolyte [42–44], ensuring a continuous supply to the microneedle array for both catalysis and monitoring (Fig. 1a, g). The surface morphology of the microneedle array was characterized using SEM. In the front view, the microneedle length of 500.0 μm is clearly visible, while in the top view, the needle tips reveal a hole structure (Fig. 1h). These holes are connected to the base of the microneedle array, facilitating gas exchange (Figs. 1i and S12). To evaluate the biocompatibility of the microneedle array, we conducted an SEM elemental distribution analysis. The results show that the array was primarily composed of C, N, and O (Fig. 1j, k). Additionally, cytotoxicity tests on the array indicated no significant cell death, demonstrating its compatibility with biological systems (Fig. S13). The microneedle array not only exhibited good material exchange capability but also good biocompatibility, indicating its promising potential for applications in portable hydrogen release and NO exchange platforms.

Subsequently, we integrated the microneedle array, electrolyte, and dual-channel electrocatalytic flexible electrode into a dual-channel electrocatalytic flexible system (Pd-Ni_5_P_4_/DCEFS). The detailed procedure included the following steps. First, the microneedle array, which was loaded with the electrolyte, was fixed onto the DCEF platform using a biopolymer sol (Fig. 1a). Next, small holes were created near the base of the microneedle array using a fine needle (Figs. 1j and S14), allowing real-time replenishment of the electrolyte. Finally, medical cotton was used to seal the holes, prevent electrolyte leakage, and ensure a continuous supply of electrolyte for stable system operation. Before encapsulating the microneedle array, we used SEM to analyze channels A and B using DCEF electrodes fabricated through a catalytic ink spin-coating process (Figs. 1e and S15). The SEM results clearly show the carbon substrate of DCEF and Pd-Ni_5_P_4_ loaded onto it, verifying the fabrication of Pd-Ni_5_P_4_/DCEFS.

When viewed from the front, the integrated Pd-Ni_5_P_4_/DCEFS shows that the microneedle array effectively covered the A, B, reference, and counter electrodes (Fig. 1j). This design ensures a continuous supply of electrolyte, enabling proper functioning of the entire catalytic circuit. In the side-view image, the microneedle array was bonded to the DCEF platform by the biopolymer sol (Fig. S16), guaranteeing the structural stability and functional integrity of the system. Notably, the hole structure of the microneedle array allows painless penetration of the skin, facilitating the localized release of hydrogen gas at the disease site, while simultaneously monitoring the endogenous NO released from the wound [45, 46]. This design endows the Pd-Ni_5_P_4_/DCEFS not only with the capability for hydrogen release and catalysis but also with the ability to monitor NO exchange in real time, thus fulfilling the dual needs for both disease site monitoring and treatment at the device design level.

### Physical Properties of Pd-Ni_5_P_4_/DCEFS

**Fig. 2:**
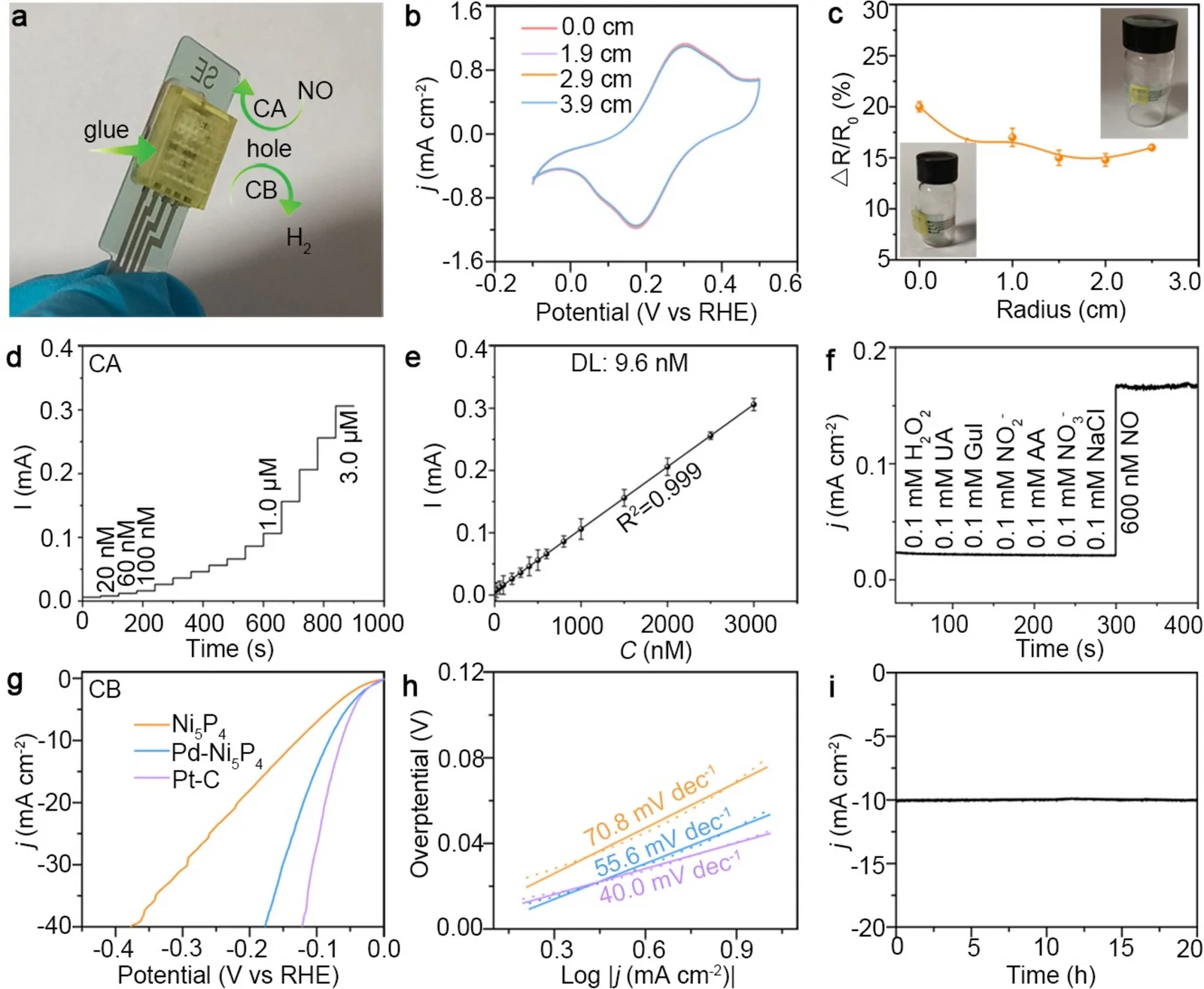
Electrochemical performance of the Pd-Ni_5_P_4_/DCEFS for dual-functional NO sensing and HER. **a** Schematic diagram of NO ingress and hydrogen evolution in DCEFS. **b** Changes in the CV curve of the system at varying bending deformation levels after loading the PBS solution into the DCEFS. **c** ΔR/R0 as a function of the curvature radius. Error bars represent the standard deviation of four independent measurements (*n* = 4, mean ± SD). CA: Performance of Pd-Ni_5_P_4_/DCEFS in electrocatalytic NO oxidation. **d** Time-dependent current response of the sensor in NO solution with varying concentrations (detection range: 20 nM to 3.0 μM) at a bias voltage of 0.75 V. **e** Calibration curve: linear relationship between response currents and NO concentrations. **f** Current response to the addition of a series of potentially interfering chemicals (0.1 mM) and NO (600.0 nM); CB electrochemical HER performance. **g** Linear sweep voltammograms acquired at a scan rate of 2.0 mV s^−1^ in PBS (pH 7.4) for the Ni_5_P_4_/DCEFS, Pt/CC, and Pd-Ni_5_P_4_/DCEFS. **h** Tafel plots of Ni_5_P_4_/DCEF, Pt/CC, and Pd-Ni_5_P_4_/DCEFS. **i** Electrocatalytic HER stability measurement for Pd-Ni_5_P_4_/DCEFS at a static overpotential of − 90.0 mV (vs RHE) for 20.0 h

The microneedle design allows for effective skin penetration, whereas the hole structure enables the release and exchange of NO and hydrogen, enhancing the operability and practicality of the system in biological environments (Fig. 2a). To investigate whether the Pd-Ni_5_P_4_/DCEFS can conform to the skin, we subjected the system to bending tests. Even under a strain of 2.5 cm, the system could return to its original state without any fracture of the microneedle array or DCEF (Fig. S17a) [47]. When the Pd-Ni_5_P_4_/DCEFS loaded with the electrolyte was connected to a light-emitting diode (LED), the circuit was closed and the LED remained lit, indicating that the path for electron flow within the system network was continuous (Fig. S17b). This ensures the stability of the electrochemical analysis and catalytic reactions.

Cyclic voltammetry (CV) tests conducted in K_3_[Fe(CN)_6_] revealed a distinct and reversible redox peak at − 0.1 and 0.5 V, demonstrating the excellent electron transferability of the Pd-Ni_5_P_4_/DCEFS (Fig. 2b). Further electrochemical stability assessments using K_3_[Fe(CN)_6_] showed that the Pd-Ni_5_P_4_/DCEFS exhibited high reproducibility in both potential and peak current across varying bending radii (1.5–3.9 cm). To evaluate the flexibility of the system, Pd- Pd-Ni_5_P_4_/DCEFS was wrapped around cylindrical objects with varying curvatures [39, 48]. By reducing the curvature radius to as small as 1.5 cm, the relative resistance change (Δ*R*/*R*_*0*_) caused by bending showed no significant variation (Figs. 2c and S18). The excellent electrochemical activity of Pd-Ni_5_P_4_/DCEFS under bending conditions enabled its conformal attachment to the mouse skin.

### System Performance Evaluation

The preparation process began with the formulation of the catalyst ink, which was prepared by dispersing 5.0 mg of the Pd-Ni_5_P_4_ catalyst and 0.5 mg of carbon black in a 1 mL mixed solvent (water/ethanol = 4:1, v/v) containing 20 μL of Nafion solution (5 wt%). Subsequently, 5 μL of the resulting ink was drop-casted onto the surface of a glassy carbon electrode (3 mm in diameter) [41, 49, 50]. Based on these specific loading parameters, the mass loading of the catalyst on the electrode was calculated to be approximately 0.35 mg cm^−2^. The active sites of the electrode were quantitatively evaluated by measuring the electrochemical double-layer capacitance (*C*_dl_), which is directly proportional to and thus used as a proxy for the electrochemical active surface area (ECSA). Cyclic voltammetry (CV) curves were recorded at various scan rates within the non-catalytic double-layer region from 0.025 to 0.12 V (vs. RHE). By fitting the linear relationship between the capacitive current density (∆ *j*/2) at 0.07 V versus RHE and the scan rate, the *C*_dl_ of Pd-Ni_5_P_4_/DCEFS was determined to be approximately 5.63 mF cm^−2^ (Fig. S19). These quantitative findings collectively demonstrate that Pd-Ni_5_P_4_ possesses a significantly enhanced electrochemical active surface area (ECSA), which provides a higher density of redox-active sites. This structural advantage serves a dual purpose: it effectively reduces the overpotential for the HER while simultaneously providing abundant contact sites for the catalytic oxidation/reduction of NO.

#### NO Sensor

To evaluate the electrochemical performance of Pd-Ni_5_P_4_/DCEFS for NO detection, we focused on the sensor response of channel A (CA; only the A-electrode clamp) to NO. The experimental results showed that, in cell culture medium, the current response to NO exhibited a good linear relationship in the range of 10.0 nM to 3.0 μM, with a high sensitivity of 1.0 μA nM^−1^ cm^−2^. Additionally, the detection limit of the electrode was as low as 9.6 nM (0.75 V, S/N = 3) (Fig. 2d, e), demonstrating the excellent performance of Pd-Ni_5_P_4_/DCEFS. More importantly, the Pd-Ni_5_P_4_/DCEFS electrode did not show any significant passivation, and the nutrients in the cell culture medium did not notably affect the electrode performance. This indicates that the Pd-Ni_5_P_4_/DCEFS electrode can maintain good electrochemical stability and efficient NO-sensing capability in complex environments.

In terms of performance comparison, the detection limit of the Pd-Ni_5_P_4_/DCEFS electrode (9.6 nM) is comparable to those of other reported electrochemical NO sensors, such as N-G/FePc/Nafion/PLL ITO (180.0 nM) [51], Au NTs/TiO_2_ NWs/Au NTs/PDMS (16.0 nM) [52], and (TTBA-rGO)/ZnO (5.0 nM) [53]. Moreover, its sensitivity (1.0 μA nM^−1^ cm^−2^, Fig. 2d, e) is significantly higher than those of several known sensors, including Ni–N_4_ (0.4306 nA nM^−1^ cm^−2^) [15], TiO_2_/CdS (0.0234 nA nM^−1^ cm^−2^) [54], AuNPs-ctDNA-NGS/SPCE (0.8609 nA nM^−1^ cm^−2^) [55], and Li_1+x_FePO_4_ (0.6492 nA nM^−1^ cm^−2^) [56]. These data indicate that Pd-Ni_5_P_4_/DCEFS possesses excellent NO catalytic oxidation performance and demonstrates strong competitiveness in NO sensing (Table S2).

To further assess the selectivity of Pd-Ni_5_P_4_/DCEFS, we tested its interaction with various interfering substances, including nitrite (NaNO_2_), glucose (C_6_H_12_O_6_), ascorbic acid (AA), urea (UA), sodium nitrate (NaNO_3_), potassium chloride (KCl), sodium chloride (NaCl), and hydrogen peroxide (H_2_O_2_) (Fig. 2f). The results indicate that only NO caused a significant change in the current response, whereas the injection of other interfering substances did not produce notable current responses. Therefore, Pd-Ni_5_P_4_/DCEFS has excellent selectivity for NO detection. As shown in Fig. 2f, when high concentrations of interfering substances (such as 0.1 mM H_2_O_2_, NO_2_^−^, NO_3_^−^, AA, UA, and glucose) were introduced, the changes in current were negligible. By contrast, when a low concentration (600.0 nM) of NO was injected, the change in current significantly increased. This further confirmed the excellent selectivity and anti-interference capability of Pd-Ni_5_P_4_/DCEFS for NO detection [57].

To test the long-term stability of the sensor, we examined and recorded the impact of the material stability of Pd-Ni_5_P_4_/DCEFS on the detection limit every 4 days (Fig. S20a). During the measurements, Pd-Ni_5_P_4_/DCEFS was stored in a dry environment at 18.0 °C. The results showed that the detection limit did not change significantly (relative standard deviation (RSD) = 0.9%), indicating that the sensor exhibited excellent long-term stability. To assess the operational stability of the sensor, a concentration-dependent NO test was conducted. In this test, 100.0 nM NO was added to the system, and the current changes were monitored (Fig. S20b). To further validate the operational stability, multiple cyclic tests were performed, with the detection limit tested every five cycles. The results showed no significant change in the detection limit, indicating that the system exhibited excellent operational stability for NO detection.

To simulate complex bioanalytical environments, an appropriate concentration of serum was incorporated into the culture medium to introduce electrochemical interferences. Experimental results demonstrate that the Pd-Ni_5_P_4_/DCEF electrode exhibits exceptional performance at an applied bias of 0.75 V: its linear detection range was extended to 30 nM-3.0 μM, with a limit of detection (LOD) as low as 14.5 nM (Fig. S21). Notably, the electrode achieved a high sensitivity of 0.75 μA nM^−1^ cm^−2^, significantly outperforming most previously reported sensors (Table S2). This underscores the superior catalytic oxidation performance of the electrode toward NO, even within complex matrices containing serum, demonstrating its robust competitive advantage in the field of real-time monitoring of trace-level NO. When the A channel was set alone, Pd-Ni_5_P_4_/DCEFS demonstrated not only a low detection limit and high sensitivity in electrochemical NO detection but also superior stability and anti-interference performance in practical applications, proving its potential in biosensors and related fields.

Pd-Ni_5_P_4_/DCEFS was used to detect the release of NO molecules from RAW264.7 cells by monitoring amperometric responses at a constant potential of 0.75 V versus Ag/AgCl in the cell culture medium. As one of the selected model cell lines, RAW264.7 was cultured on the sensor surface for 12.0 h. L-Arg treatment activates the NOS pathway, leading to the production of endogenous NO in live cells [58, 59]. We investigated the ability of Pd-Ni_5_P_4_/DCEFS to monitor the release of endogenous NO from RAW264.7 cells under L-Arg stimulation (Fig. S22). Treatment with 10 mM l-Arg resulted in a significant increase in the oxidation current. Moreover, when RAW264.7 cells were simultaneously treated with 10 mM L-Arg and 10 mM L-NG-nitroarginine methyl ester (L-NAME, a precursor of the eNOS inhibitor), no increase in current was observed. This clearly confirmed that the observed current response was due to the detection of extracellular endogenous NO by Pd-Ni_5_P_4_/DCEFS. These results demonstrate that Pd-Ni_5_P_4_/DCEFS can detect NO released from live cells.

#### Electrocatalytic HER Measurements (Channel B)

To further evaluate the electrochemical HER performance of Pd-Ni_5_P_4_/DCEFS, we used channel B (CB) of the DCEF electrode. In the experiment, we used phosphate-buffered saline as the electrolyte and employed linear sweep voltammetry (LSV) to obtain polarization curves, which were then used to estimate the electrochemical catalytic activity of Ni_5_P_4_/DCEFS, Pd-Ni_5_P_4_/DCEFS, and Pt − C/carbon cloth (CC) in the HER. The experimental results showed that the Pd-Ni_5_P_4_/DCEFS required only − 91.0 mV of overpotential to reach a standard current density of − 10 mA cm^−2^, which is considerably close to the performance of Pt-C/CC (− 50.2 mV, Fig. 2g). This result indicates that Pd-Ni_5_P_4_/DCEFS exhibits excellent catalytic activity under neutral conditions, overcoming the performance bottleneck of the neutral electrocatalysts (Table S3).

To compare the catalytic activities of the active sites in Pd-Ni_5_P_4_/DCEFS, we used the estimated turnover frequency (TOF) [60, 61]. Assuming that the HER is a single-electron process, we calculated the upper limit of the number of active sites (Fig. S23). Under the condition of pH = 7.4, after normalizing the polarization curve, the TOF was 0.51 s^−1^, and the overpotential of the electrode was only 100 mV. This result indicates that each active site of Pd-Ni_5_P_4_/DCEFS demonstrates exceptional catalytic ability, demonstrating its superior performance in the HER. The Pd-Ni_5_P_4_/DCEFS performance in the HER is comparable to that of Pt − C/CC, and its outstanding catalytic efficiency and low overpotential make it an ideal catalyst for hydrogen evolution under neutral conditions.

The Tafel slope is an important indicator for assessing the kinetics of catalytic reactions. A smaller Tafel slope indicates a faster rate of change in current density with respect to voltage, indicating that the catalytic reaction responds more quickly [62, 63]. In this experiment, the Tafel slope of Pd-Ni_5_P_4_/DCEFS was 55.6 mV dec^−1^, which is close to the value for Pt − C (40.0 mV dec^−1^) (Fig. 2h). This suggests that Pd-Ni_5_P_4_/DCEFS exhibits faster reaction kinetics in the HER and excellent catalytic efficiency compared with traditional catalysts. Further tests revealed that Pd-Ni_5_P_4_/DCEFS demonstrates outstanding electrochemical stability for the HER. In a 20-h steady-state current test, the electrode exhibited a stable current response (Fig. 2i).

Multiple cyclic tests were conducted to verify stability. First, after performing LSV tests in phosphate-buffered saline (PBS) solution, the electrode was rinsed with deionized water, dried in an environment at 50 °C, and then re-immersed in PBS for HER performance testing. Even with an increased number of test cycles, the overpotential change of the Pd-Ni_5_P_4_/DCEFS was minimal (Fig. S24), indicating its strong stability and durability. After 200 and 600 CV tests, the electrode overpotential barely changed, further confirming the electrochemical stability and excellent performance of Pd-Ni_5_P_4_/DCEFS during long-term use.

Notably, the faradaic efficiency was estimated by comparing the amount of hydrogen gas produced during constant-potential cathodic electrolysis with the amount of hydrogen gas calculated theoretically based on electrolysis (assuming 100% faradaic efficiency) [64]. The experimental results at pH 7.4 show near 100% faradaic efficiency for hydrogen evolution (Fig. S25), indicating that Pd-Ni_5_P_4_/DCEFS efficiently utilizes current in the HER. According to Faraday’s Law of Electrolysis, the amount of hydrogen (n_H2_) is directly proportional to the total electric charge (Q) passed through the electrode. The calculation uses the formula: n_H2_ = Q/zF = It/zF [49]. Where I is the HER current, t is the duration (35.0 min), z is the number of transferred electrons (z = 2 for 2H^+^ + 2e^-^ → H_2_), and F is the Faraday constant (96,485 C mol^−1^). At a potential of − 0.25 V vs. RHE, based on the electrode area (5.0 mm diameter), the theoretical hydrogen yield for a 35-min treatment cycle was calculated to be 149.6 μmol. To verify the actual output, we employed the water displacement method [49]. Experimental results showed an actual yield of 147.4 μmol at -0.25 V (Fig. S26). By comparing the theoretical and experimental values, the Faraday Efficiency (FE) of the system (different potentials) was determined to be 98.5%. This indicates that nearly all the applied current is utilized for therapeutic hydrogen production, ensuring precise and controllable dosing. To further analyze the catalytic performance of Pd-Ni_5_P_4_/DCEFS in the HER, we employed electrochemical impedance spectroscopy (EIS) to characterize the interfacial charge-transfer process (Fig. S27) [65]. The experimental results show that the charge-transfer resistance (*Rct*) of Pd-Ni_5_P_4_/DCEFS is similar to that of Pt-C/CC, suggesting that Pd-Ni_5_P_4_/DCEFS has a rapid charge-transfer rate during the HER, and its catalytic activity is not significantly influenced by the substrate.

We used XRD and TEM to further explore the catalytic mechanism of the Pd-Ni_5_P_4_/DCEFS surface for the HER. No significant residual phase peaks were observed after electrochemical activation compared with the initial state, indicating that the crystal structure of the catalyst did not undergo noticeable changes during the electrochemical activation process (Fig. S28). Furthermore, activated Pd-Ni_5_P_4_/DCEFS retained its original irregular sheet-like structure and composition, indicating that the electrochemical activation process did not damage the basic structure or performance of the catalyst (Fig. S29). In situ electrochemical infrared spectroscopy indicated that the introduction of Pd altered the behavior of the catalyst toward H_2_O adsorption (Fig. S30). Specifically, at low potentials, Ni first adsorbs H_2_O; as the potential increases, both Pd and Ni cooperatively adsorb H_2_O, overcoming the challenges of H_2_O adsorption and the formation of M-*H_2_O under neutral conditions [66, 67]. During this process, the generated *H_2_O dissociates into *H, which binds to Pd sites, facilitating hydrogen production. Therefore, the introduction of Pd significantly enhances the catalytic performance, primarily owing to its ability to facilitate the adsorption and transformation of *H_2_O [36, 68, 69]. This synergistic interaction between Pd and Ni effectively lowers the energy barrier for water dissociation, thereby accelerating the neutral HER kinetics.

The stability tests for HER catalysis and NO indicated that, after multiple cycles, the system maintained its stability and excellent detection and catalytic performance (Fig. S31). To further evaluate the chemical stability and electrochemical durability of Pd-Ni_5_P_4_/DCEF in biological culture media-specifically to assess its service life within complex bio-environments-the electrode was immersed in the culture medium for 10 days, with its electrochemical performance monitored every 48 h. As illustrated in Fig. S32, the initial overpotential was − 92.0 mV; after the 10-day immersion period, it exhibited only a negligible fluctuation to − 92.5 mV, representing a total increase of less than 0.54%. Simultaneously, the limit of detection (LOD) of the sensor demonstrated exceptional stability (Fig. S32), remaining consistently at approximately 14.5 nM throughout the monitoring cycle without any significant performance degradation. These results provide robust evidence that Pd-Ni_5_P_4_/DCEF possesses outstanding long-term stability and structural durability within complex culture media. The excellent catalytic performance and stability of Pd-Ni_5_P_4_/DCEFS, supported by these characterization results, demonstrate its potential for practical applications while ensuring system reliability for biological research.

### Channel A in Pd-Ni_5_P_4_/DCEFS for Teal-Time Monitoring of NO Released by Cells

The mouse macrophage cell line RAW264.7, derived from mouse mononuclear macrophages, is widely used to study inflammatory mechanisms and immune responses [70]. Owing to its high sensitivity to various stimuli such as bacteria, viruses, and other inflammatory factors, it plays an irreplaceable role in anti-inflammatory treatment research. First, we assessed the cytotoxicity of Pd-Ni_5_P_4_/DCEFS using a Cell Counting Kit-8 (CCK-8). Even with prolonged incubation times, the survival rate of RAW264.7 cells remained high, demonstrating that Pd-Ni_5_P_4_/DCEFS has excellent biocompatibility (Figs. S33 and S34). In physiological solutions, Pd-Ni_5_P_4_/DCEFS demonstrated excellent performance in monitoring NO release. To further explore the ability of Pd-Ni_5_P_4_/DCEFS to monitor NO release during cellular polarization, we selected channel A of Pd-Ni_5_P_4_/DCEFS for sensitive detection, focusing on changes in NO levels under cellular polarization states.

**Fig. 3:**
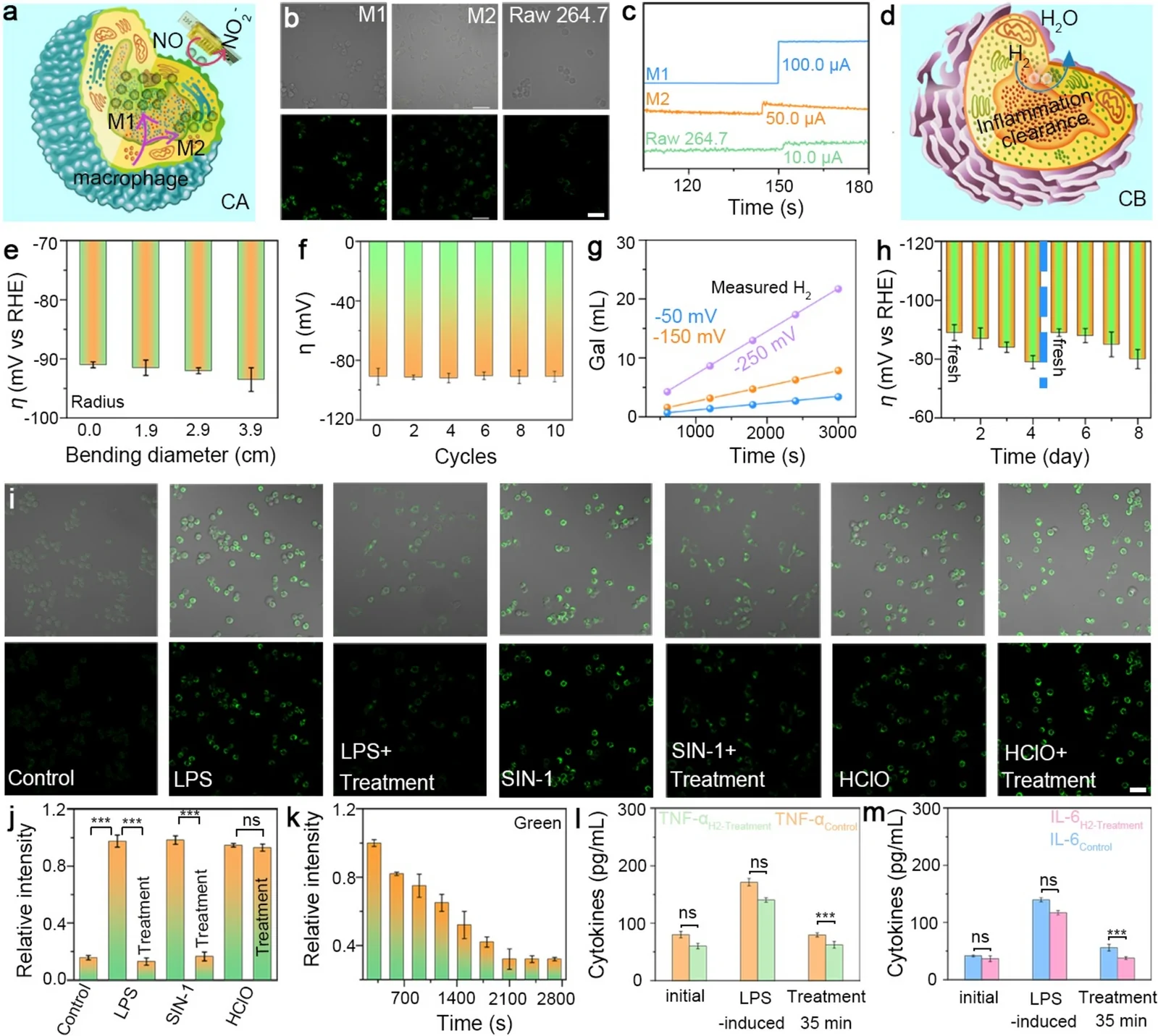
In vitro validation of the Pd-Ni_5_P_4_/DCEFS system for real-time monitoring of cell-derived NO and H_2_-mediated cellular anti-inflammatory therapy. CA: **a** Schematic diagram for monitoring the release of endogenous NO. **b** Microscopic images of the initial state (RAW264.7 cells), LPS induction (M1-type cells), and 35 min of hydrogen gas treatment (M2-type cells). Scale bars: 50 μm. **c** Monitoring NO release from RAW264.7 cells, LPS-induced M1-type cells, and M2-type cells after hydrogen gas treatment. CB: **d** Schematic of hydrogen gas clearing inflammation. **e** Comparison of the hydrogen gas evolution overpotential for varying bending degrees of the system. **f** Pd-Ni_5_P_4_/DCEFS exhibits superior catalytic stability after multiple CV cycles. **g** Quantification of H_2_ gas produced by Pd-Ni_5_P_4_/DCEFS using the drainage method at varying voltages and comparison of the corresponding results with theoretical values. **h** Relationship between time and overpotential after the addition of an electrolyte. **i** Monitoring the variations in the levels of various reactive species (·OH, ONOO–, and HClO) in cells using confocal laser scanning microscopy images in the presence and absence of H_2_ gas anti-inflammatory treatment. Scale bars: 50 μm. **j** Selection of optimal H_2_ gas anti-inflammatory treatment conditions and analysis of the elimination effects of different reactive species by assessing changes in the relative intensity of green fluorescence. **k** Gradual extension of the H_2_ evolution time and assessment of the removal effect of ·OH via evaluation of the changes in the relative intensity of green fluorescence. **l, m** Change curves of inflammatory factors in the initial state, after LPS induction, and after 35 min of hydrogen gas treatment (*n* = 4, mean ± SD). Statistical significance was calculated using one-way ANOVA with Tukey’s multiple comparison test; ns: no significance, *p < 0.05, **p < 0.01, and ***p < 0.001

To verify the application of Pd-Ni_5_P_4_/DCEFS under different macrophage polarization states, we first placed the electrode system in the cell culture medium and measured the baseline current at a voltage of 0.75 V. After 12 h of culturing RAW264.7 macrophages, the current was measured again (green line) to obtain the baseline NO level under untreated conditions. Next, to induce macrophage polarization, interferon-γ (IFN-γ) and lipopolysaccharide (LPS) were added, and the cells were co-cultured for 24 h to induce M1 polarization (Fig. 3a, b). At this stage, the current significantly increased at 0.75 V (blue line), indicating an increase in the NO concentration. Subsequently, the cells were co-cultured with interleukin-4 (IL-4) to induce M2 polarization, which resulted in a noticeable decrease in NO release (orange line) (Fig. 3a, c). These results clearly show significant differences in NO release between macrophages in different polarization states. When macrophages were polarized to the M1 type, the NO concentration increased, and the corresponding current signal was significantly elevated, indicating an active inflammatory state. By contrast, when macrophages were polarized to the M2-type, the NO concentration decreased and the current signal significantly decreased, confirming that inflammation was resolved [16]. Therefore, Pd-Ni_5_P_4_/DCEFS holds great potential for real-time monitoring of NO release in macrophage subtypes, providing an important biomarker for studying skin wound healing and inflammation resolution and offering strong support for understanding the dynamics of the inflammatory environment.

### Hydrogen Evolution in Channel B of Pd-Ni_5_P_4_/DCEFS for Intracellular Inflammation Clearance

The Pd-Ni_5_P_4_/DCEFS electrode demonstrated excellent electrocatalytic hydrogen evolution performance in physiologically relevant solutions. We first selected the B channel of the electrode to evaluate its hydrogen-based anti-inflammatory effect, utilizing the reducing properties of hydrogen to effectively eliminate the related oxidative species in the cells (Fig. 3c, d). Furthermore, during real-time hydrogen-generation experiments using the Pd-Ni_5_P_4_/DCEFS via the I − T method, cell death was not significant, further confirming the system’s good biocompatibility (Fig. S34b). To further investigate the performance stability of Pd-Ni_5_P_4_/DCEFS under bending conditions, we subjected the electrode to different bending radii (0, 1.9, 2.9, and 3.9 cm). No significant changes in the overpotential were detected during the tests, indicating that the catalytic performance of Pd-Ni_5_P_4_/DCEFS remained unaffected, even when bent or deformed (Fig. 3e). Under bending conditions, Pd-Ni_5_P_4_/DCEFS maintained its excellent catalytic stability after multiple CV cycles (Fig. 3f). This ensures efficient hydrogen evolution when applied to living skin, thereby providing reliable support for device performance.

To study the relationship among voltage, time, hydrogen production efficiency, and inflammation clearance, we monitored the hydrogen evolution over time (600–2100s) at varying constant voltages (from − 0.1 to − 0.3 V) (Fig. 3g). The hydrogen production efficiency was consistent with the theoretical values, ensuring continuous hydrogen release in physiological environments. To further evaluate the relationship between hydrogen evolution and the electrolyte in the Pd-Ni_5_P_4_/DCEFS, we simulated an in vivo testing environment. After testing, the system was left in air. Over time, the overpotential decreased, which may be related to the overpotential fixed within the microneedle array (Fig. 3h). Notably, when the electrolyte was replenished, the overpotential returned to its original value, demonstrating that supplementing with fresh electrolyte every 4 days ensures normal system operation.

The active species in cells primarily include reactive oxygen species (ROS) and reactive nitrogen species (RNS) such as the superoxide anion (O_2_^−^), hydrogen peroxide (H_2_O_2_), hydroxyl radical (·OH), hypochlorous acid (HClO), singlet oxygen (^1^O_2_), nitric oxide (NO), nitrite ion (NO_2_^−^), nitrogen oxide radicals (HNO), and peroxynitrite (ONOO-). These active species play crucial roles in cellular signal transduction, immune responses, antimicrobial activity, and cellular damage [54, 55]. To identify the active species involved in the action of hydrogen in cells, we selected the most representative oxidative species in cells: ·OH, ONOO-, and HClO.

To validate the selective ROS scavenging capability of hydrogen, an in-situ electrochemical monitoring platform was developed based on a dual-channel electrode setup. An in-situ electrochemical monitoring system was established using a dual-channel electrode configuration. Channel A (the catalytic electrode) was modified with Pd-Ni_5_P_4_ nanosheets and held at a hydrogen evolution potential of − 0.1 V vs. RHE to generate H_2_. Channel B (the detection electrode), a carbon-based electrode, was maintained at a constant reduction potential of + 0.2 V versus Ag/AgCl for real-time monitoring of concentration fluctuations of oxidative species in the electrolyte. To evaluate the selective scavenging efficacy against highly reactive radicals (∙OH and ONOO^−^) and other oxidants (e.g., HClO), the electrolyte was spiked with Fenton’s reagent, an acidified nitrite reaction system, and commercial HClO solution, respectively (Fig. S35). Upon the acquisition of a stable characteristic reduction current at Channel B, Channel A was activated to generate H_2_, and the subsequent current response at Channel B was recorded to quantify the scavenging impact. Subsequently, the status of the electrolyte was restored by deactivating Channel A to observe the recovery of the reduction current, thereby eliminating potential baseline drift and confirming the direct antioxidant role of H_2_.

To extend the findings from the electrochemical model to biological contexts, the scavenging efficacy of hydrogen against different ROS/RNS components was further evaluated in vitro using cell-based assays. In the control group, untreated cells were incubated only with DCFH-DA for imaging, and weak fluorescence signals were observed, indicating a low level of ROS/RNS in the cells. LPS, commonly used to induce inflammation in RAW264.7, led to an excessive burst of ROS/RNS. After stimulating the cells, a significant enhancement in green fluorescence was observed. However, after hydrogen treatment, the signal was significantly reduced. In experiments using Fenton's reagent to generate ·OH, a continuous hydrogen supply substantially reduced the green fluorescence intensity, demonstrating that hydrogen effectively cleared ·OH from the cells (Fig. 3i, g, LPS and LPS + treatment). Using 3-morpholino-sydnonimine (SIN-1) as an ONOO^−^ donor, continuous hydrogen treatment of RAW264.7 cells also markedly decreased the green fluorescence intensity, indicating that hydrogen effectively cleared ONOO^−^ (Fig. 3i, g, SIN-1 and SIN-1 + treatment). HClO exhibits strong antimicrobial activity and plays a crucial role in wound healing through multiple processes, including immune regulation, anti-inflammatory effects, and tissue regeneration, thereby helping clear and control infections. Therefore, we selected HClO as the target for further investigation of the anti-inflammatory effects of hydrogen. Notably, even after adding HClO, strong green fluorescence signals persisted after hydrogen treatment, with only minor changes (Fig. 3i, g, HClO, and HClO + treatment). These results indicate that hydrogen can selectively clear ROS/RNS while preserving other essential active species (including HClO) to ensure normal intracellular signal regulation. This is consistent with the results of previous studies.

To further investigate the intrinsic relationship between green fluorescence intensity and time, we used Fenton’s reagent to induce the generation of hydroxyl radicals (·OH) within cells. A continuous hydrogen supply efficiently scavenged ·OH and significantly reduced the intensity of the green fluorescence signal. We employed 2′,7′-dichlorodihydrofluorescein diacetate (DCFH-DA) as a green fluorescence probe to detect ·OH levels, with Fenton’s reagent acting as the inducer for intracellular ·OH generation. Under a target voltage of − 0.25 V, we observed the changes in normalized fluorescence intensity by varying the duration of hydrogen supply, allowing us to assess fluctuations in the ·OH level (Figs. 3k and S36). After stimulating the cells with Fenton's reagent, a strong green fluorescence signal was detected, indicating a substantial generation of ·OH. As the hydrogen supply time was extended, the fluorescence intensity gradually decreased, and ·OH levels diminished, indicating that hydrogen effectively scavenged ·OH and exerted anti-inflammatory effects. When the hydrogen supply was extended to 2100s (35 min), the fluorescence intensity considerably reduced. Notably, extending the hydrogen supply time to 2800 s did not lead to further notable changes in the fluorescence intensity. Therefore, a 35 min hydrogen supply at − 0.25 V produced the optimal anti-inflammatory effect. These findings provide a basis for experimental studies of customized electrocatalytic hydrogen evolution in vivo, particularly for skin applications. Notably, changes in cell morphology were observed through the removal of oxidized and nitrified species or direct hydrogen-induced inflammation clearance (Figs. 3i, S36, and S37). These changes reflect the transition from an inflammatory state to resolution of inflammation, which is consistent with the shift from M1 to M2 macrophages. Therefore, changes in NO levels during the treatment process can be used to assess the degree of resolution of inflammation.

We have performed immunofluorescence (IF) staining experiments to evaluate the protein expression levels of iNOS (a characteristic marker for the pro-inflammatory M1 phenotype) and Arg-1 (a characteristic marker for the anti-inflammatory M2 phenotype). M1 Polarization (iNOS Expression): In the control group, a significantly higher fluorescence intensity of iNOS was observed, indicating a distinct M1 phenotype under oxidative stress or specific inductive conditions. In contrast, the iNOS fluorescence signal was markedly attenuated in the Pd-Ni_5_P_4_/DCEFS-treated group, demonstrating that the system effectively inhibits M1 polarization. M2 Polarization (Arg-1 Expression): Conversely, the expression of Arg-1 (green fluorescence) was significantly upregulated in the experimental group compared to the control. The merged images clearly illustrate a robust phenotypic transition within the macrophage population-shifting from a pro-inflammatory M1 state toward a pro-healing M2 state under our treatment (Fig. S38). We evaluated the effect of the Pd-Ni_5_P_4_/DCEFS on inflammatory factors by measuring the changes in the concentrations of 3-NT (3-nitrotyrosine), IL-1β (interleukin-1 beta), IL-6 (interleukin-6), and TNF-α (tumor necrosis factor-alpha) in the cells. Under the initial conditions, their levels were within the normal range, which is consistent with previous studies [73]. Upon stimulation with LPS, the levels of these inflammatory factors significantly increased. However, after 35 min of hydrogen treatment, these inflammatory factors notably decreased to normal levels, further confirming the effectiveness of hydrogen in the clearance of inflammation (Fig. 3l, m).These direct lines of evidence at the protein level are highly consistent with the previously reported trends in cytokine secretion (e.g., TNF-α, IL-10), collectively confirming the potent regulatory effect of Pd-Ni5P4/DCEFS on macrophage polarization.

### Closed-Loop Dynamic Treatment Effects of Pd-Ni_5_P_4_/DCEFS at the Cellular Level

Given that channels A and B demonstrate excellent detection capabilities and hydrogen-mediated anti-inflammatory effects when used individually, we designed a combined program on an electrochemical workstation that integrates both channels. Specifically, channel A (0.75 V, 100 s) was set as the primary program, whereas channel B (− 0.25 V, 35 min) served as the backup, enabling the closed-loop dynamic treatment protocol.

**Fig. 4:**
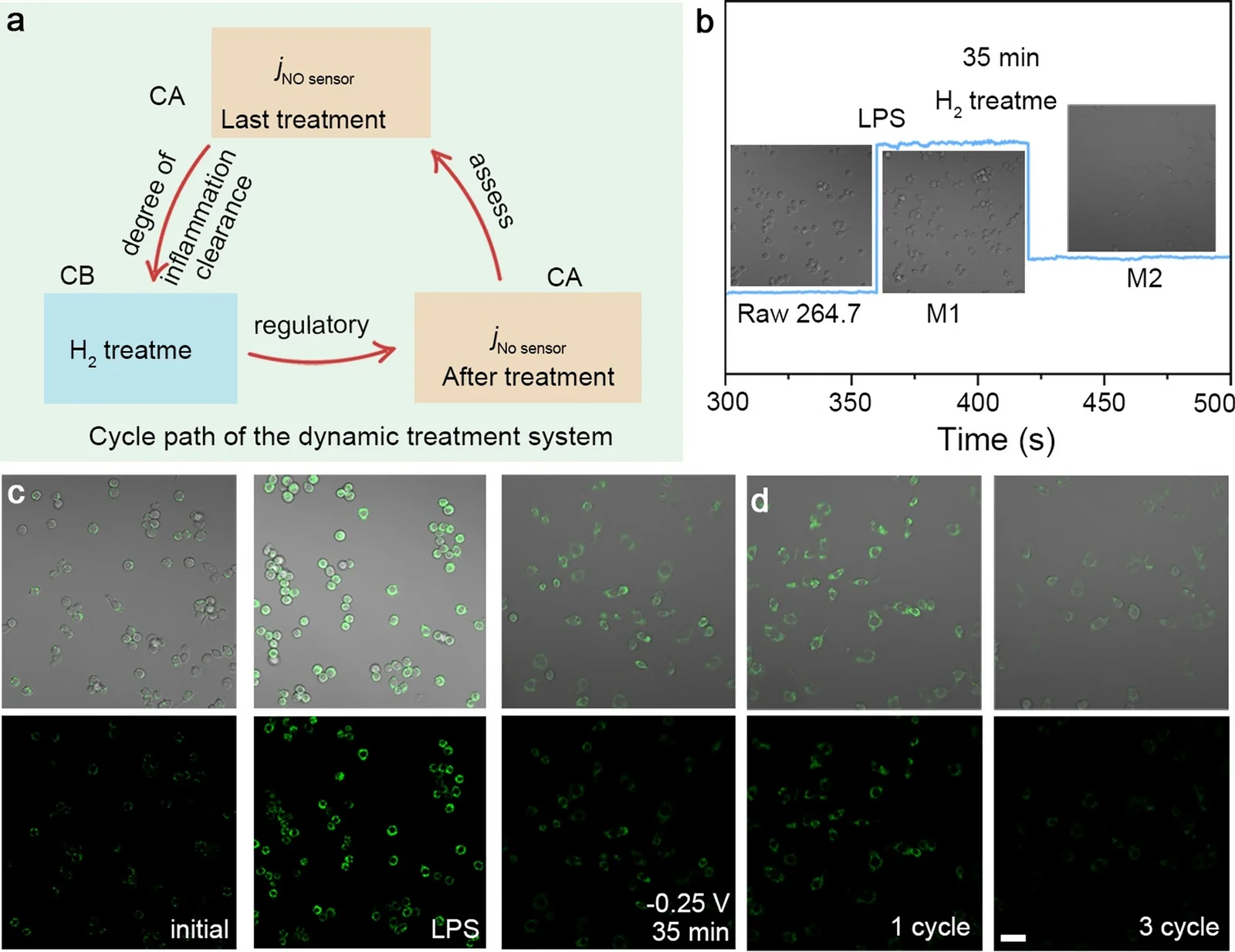
Evaluation of the dynamic and cyclic therapeutic efficacy of the Pd-Ni_5_P_4_/DCEFS system at the cellular level.** a** Schematic of a single dynamic treatment cycle. **b** Monitoring of NO release after LPS induction in M1-type cells, followed by hydrogen gas treatment to form M2-type cells. **c** Microscopic images corresponding to LPS induction and hydrogen gas treatment. **d** Intracellular inflammatory resolution/clearance efficacy after one and three cycles of system treatment following LPS stimulation (*n* = 4, mean ± SD). The error bars represent the standard deviation of four independent measurements. Scale bars: 50 μm

To improve user convenience, we introduced a single-cycle testing method (Fig. 4a). The operational steps are as follows. First, the baseline current (*j*_baseline_) is measured using channel A, which serves as the reference for all subsequent NO current changes. Then, using the dual-channel program, Pd-Ni_5_P_4_/DCEFS was placed in LPS-induced RAW264.7, and the current change (*j*_initial_) induced by NO was measured. Upon completion of the NO detection program, hydrogen began to evolve. After 35 min of hydrogen evolution, the trend of NO current change (*j*_*i*nitial_—*j*_baseline_) was observed to assess the inflammatory status. After waiting for 5–10 min, the program was re-run to measure the change in NO current (*j*_treatment_—*j*_baseline_), and a comparative analysis of the hydrogen-mediated anti-inflammatory effects was conducted. By comparing the two current changes (*j*_initial_—*j*_baseline_ and *j*_treatment_—*j*_baseline_), the effect of hydrogen on inflammation clearing can be evaluated, providing insights into the potential for wound healing. The cyclic program constituted a complete closed-loop dynamic treatment process.

During a single dynamic treatment using Pd-Ni_5_P_4_/DCEFS, a pronounced increase in current density was observed, indicating an exacerbation of inflammation (Fig. 4b). After 35 min of hydrogen treatment, a marked decrease in current density was detected, demonstrating the effective clearance of inflammation. Notably, the current density remained higher than the baseline, which was consistent with previous reports on NO release from M2-type cells [24]. After LPS stimulation, RAW264.7, cells begin to differentiate into M1-type cells. However, following hydrogen treatment, a noticeable increase in the number of M2-type cells was observed. This change corresponds to the concentration of NO released. Fluorescence imaging further revealed that after cellular stimulation, the green fluorescence signal was significantly enhanced; however, after hydrogen treatment (Fig. 4c), this signal was markedly attenuated. In LPS-stimulated RAW264.7, the fluorescence intensity progressively decreased after one cycle of closed-loop dynamic treatment, indicating the initiation of intracellular inflammatory clearance. After three cycles, the fluorescence signal markedly diminished to near-baseline levels, demonstrating a near-complete resolution of inflammation. These results indicate that three cycles of the closed-loop dynamic treatment system achieved the most effective inflammatory clearance (Fig. 4d). These experimental results were consistent with the detection and hydrogen-mediated anti-inflammatory effects observed using Channels A and B individually, confirming the excellent cellular closed-loop dynamic treatment effects of Pd-Ni_5_P_4_/DCEFS.

### Mechanical Performance and Multiaxial Skin Adhesion Durability Evaluation of Pd-Ni_5_P_4_/DCEFS Microneedle Patches

The force–displacement curves were obtained by applying compression to the microneedles. The smooth and continuous curve of DCEF indicates that no needle failure occurred under loads up to 2.0 N/needle (Fig. S39a). At a displacement of 600.0 µm, the compressive force was recorded at 1.0 N/needle, which significantly exceeds the reported minimum effective force required for skin penetration (0.045 N/needle). These results confirm the superior mechanical strength of DCEF for skin puncturing and its scalability for in vivo applications. H&E staining results demonstrated that DCEF created microchannels within the skin, with a diffusion depth exceeding 150.0 μm (Fig. S39b). These findings underscore the superior transdermal capability of the DCEF microneedle patch and its effectiveness in delivering therapeutic payloads to local skin sites [74, 75].

To further elucidate the mechanical durability of the bio-gelatin-assisted adhesive microneedle arrays under complex multiaxial skin strains, such as stretching and torsion, we conducted comprehensive validations through shear, tensile, and peeling tests (Figs. S40-S42). The patches were applied to porcine skin, and it was observed that the Pd-Ni_5_P_4_/DCEF remained securely attached without flaking or detachment (Fig. S40) even under twisting, stretching, and bending movements after the addition of phosphate-buffered saline (PBS). The adhesion performance of the patches was subsequently evaluated through lap shear, tensile, and 180° peel tests (Figs. S41 and S42). Experimental results demonstrated that the bio-gelatin-assisted microneedle arrays exhibited exceptional adhesion metrics, with a peak shear force of approximately 25 N and a tensile force of approximately 6.5 N (Fig. S41a, b). Notably, during the peel test, the interface maintained high stability over a large displacement exceeding 25 mm, with a consistent peeling force of approximately 1.6 N. The calculated interfacial toughness reached approximately 320 J m^−2^ (Fig. S41c). This outstanding energy dissipation capacity under various loading modes-including tangential twisting and normal tension-ensures the structural integrity of the system during complex dynamic deformations of the skin. This toughness is fundamentally attributed to the rearrangement of gelatin molecular chains and the reversible dissipation mechanism of physical crosslinking points. In addition, the microneedle arrays, utilizing bio-gelatin as an adhesive interface (Fig. S42), exhibit superior shear strength (250 kPa) and tensile strength (72 kPa). This robust adhesion, based on biological macromolecules, ensures that the device maintains excellent interfacial integrity during tangential twisting or normal stretching of the skin, effectively preventing detachment between the microneedles and the tissue. Collectively, these microscopic mechanisms absorb external impacts, effectively preventing mechanical failure or accidental detachment of the microneedle arrays throughout the therapeutic cycle.

### Closed-Loop Dynamic Therapeutic Effect of Pd-Ni_5_P_4_/DCEFS on Skin Wounds of Diabetic Mice

**Fig. 5:**
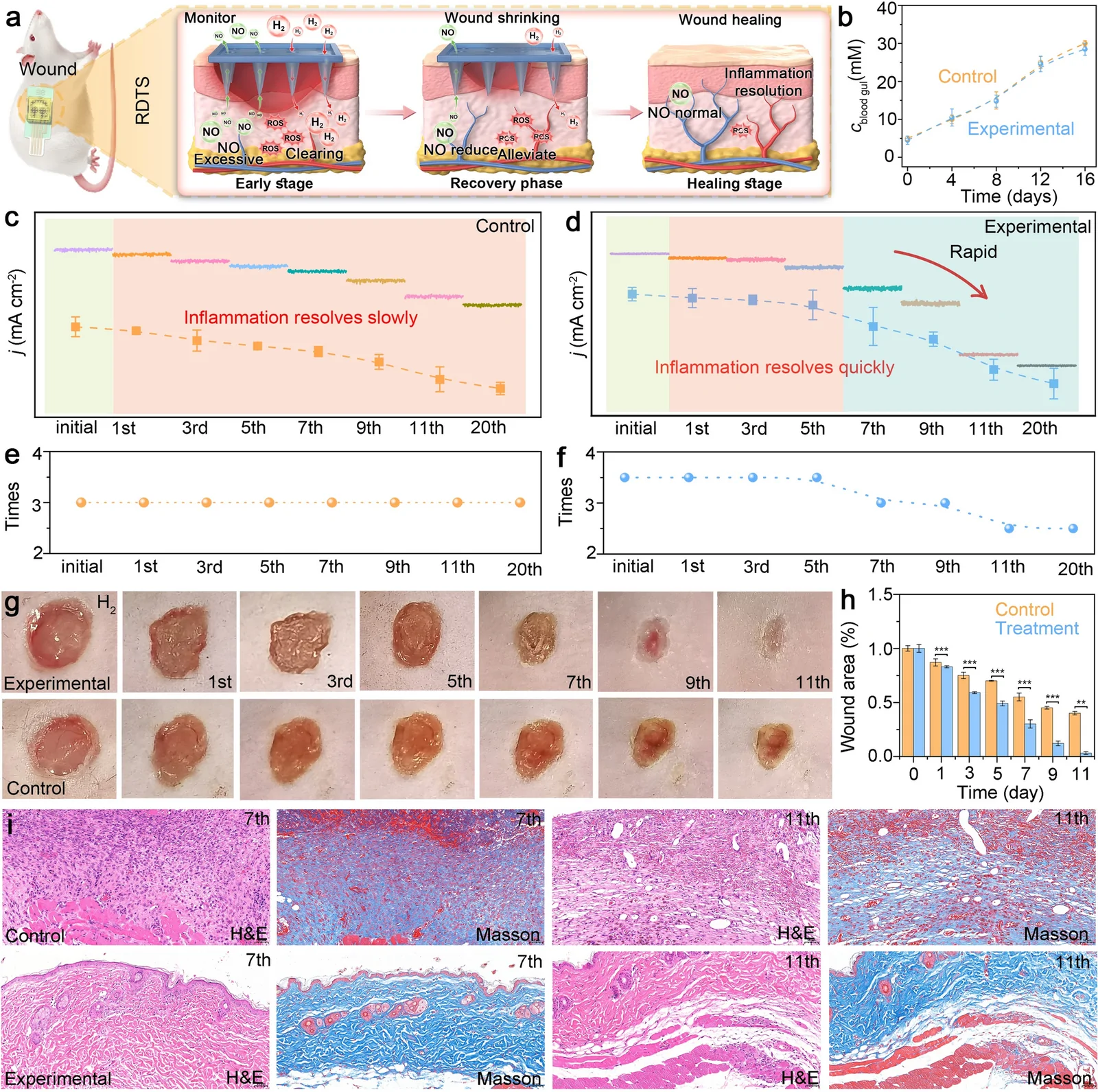
Dynamic treatment effect evaluation of the dual-channel electrocatalytic flexible system in diabetic mice. **a** Schematic diagram showing how the dual-channel electrocatalytic flexible system accelerates wound healing in diabetic mice. **b** Blood glucose concentrations in diabetic mice at varying time points after STZ injection (*n* = 4 biologically independent samples). Current change curve of NO release over time in wound monitoring with and without a dual-channel electrocatalytic flexible system. **c** Without the dual-channel electrocatalytic flexible system. **d** Dynamic treatment with the dual-channel electrocatalytic flexible system. **e** Control and **f** experimental group dynamic treatment frequency curves. The control group was tested for NO current changes three times a day without hydrogen treatment. **g** Control group: representative images of diabetic wounds after 11 day of treatment. Experimental group: Representative images of diabetic wounds after 11  day of H_2_ anti-inflammatory treatment using Pd-Ni_5_P_4_/DCEFS. **h** Relationship between wound area variation and different treatments. **i** Hematoxylin–eosin (H&E) staining (top panel) and Masson’s trichrome staining (bottom panel) of wounded skin tissues after 7 and 11 day in diabetic mice. Error bars represent the standard deviation of four independent measurements (*n* = 4, mean ± SD). Statistical significance was calculated using one-way ANOVA with Tukey’s multiple comparison test; ns: no significance, *p < 0.05, **p < 0.01, and ***p < 0.001

Based on the aforementioned cellular experimental results, we set the system’s voltage at –0.25 V and maintained it by injecting electrolyte every 2 day using a syringe. Each treatment lasted for 35 min to achieve optimal results (Fig. 5a). To assess the application potential of the Pd-Ni_5_P_4_/DCEFS in diabetic skin wound healing, we fixed the system onto the wounds of mice using tape. The tape serves to ensure a stable connection between the system and adapter. On this basis, we established a diabetic mouse model by intraperitoneally injecting streptozotocin (STZ), which damages pancreatic β-cells and raises the blood glucose level (Fig. 5b) [24]. Fourteen days after STZ injection, the blood glucose level exceeded 20 mM, and a 5 mm diameter circular wound was created on the backs of the mice.

The foreign body response elicited by Pd-Ni_5_P_4_/DCEFS is a critical factor in evaluating its biocompatibility. Following microneedle implantation, neutrophils and macrophages typically infiltrate the wound site, which can subsequently induce a fibrotic response. To assess the immunomodulatory capacity of Pd-Ni_5_P_4_/DCEFS, we performed immunofluorescence staining of CD68 (a macrophage marker) and MPO (a neutrophil marker) in tissue samples on day 10 post-surgery (Fig. S43). The results demonstrated significant infiltration of CD68⁺ and MPO⁺ cells in the control group, indicating a severe inflammatory response. In contrast, the levels of these inflammatory markers were significantly reduced in the Pd-Ni_5_P_4_/DCEFS group, with almost no evident inflammatory aggregation observed. These findings suggest that Pd-Ni_5_P_4_/DCEFS effectively suppresses the innate inflammatory response by establishing an ideal microenvironment for wound healing, thereby demonstrating its superior biocompatibility.

In addition to evaluating cellular responses in tissue, we also quantified the systemic and local levels of key inflammatory factors and oxidative stress indicators over time. First, to evaluate the effect of the Pd-Ni_5_P_4_/DCEFS on inflammatory factors, we collected blood samples from the wound area and measured the levels of 3-nitrotyrosine (3-NT) and interleukin-1β (IL-1β). The sample collection times were 1, 3, 5, 7, 9, and 11 day. The experimental results showed that, compared with day 1, the IL-1β level was significantly reduced on day 11, indicating that the system effectively alleviated the inflammatory response. Meanwhile, the 3-NT level peaked on day 3 and returned to baseline levels on day 11, reflecting the dynamic fluctuation of ONOO^−^ levels during wound healing (Fig. S44). Additionally, the levels of inflammatory cytokines such as tumor necrosis factor-α (TNF-α) and IL-6 showed a marked increase during wound healing, followed by a gradual decrease to low inflammatory levels over time, further confirming the effectiveness of the Pd-Ni_5_P_4_/DCEFS in reducing inflammation and promoting wound healing.

Next, we evaluated the dynamic therapeutic potential of the Pd-Ni_5_P_4_/DCEFS in accelerating diabetic skin wound healing, focusing on its ability to promote wound healing by clearing inflammation. During treatment, a dynamic therapeutic system was applied three times daily. The therapeutic effect was assessed by recording and comparing the NO current density curves before and after treatment and plotting the changes in the NO current. The results from the control group showed a slow decline in NO current between days 1 and 11, and the scabbing and wound-closure processes were similarly slow. Even by day 11, the wound had not fully closed and the NO current did not appreciably decrease, indicating that inflammation had not been effectively cleared. By day 20, the wound was still not fully closed and the NO current remained unchanged, further confirming that inflammation persisted (Fig. 5c, e, g, h).

By contrast, mice treated with the Pd-Ni_5_P_4_/DCEFS showed a significant improvement. Starting on day 5, the NO current in the treatment group notably decreased, indicating that the inflammatory response was effectively cleared (Fig. 5d). To observe dynamic therapeutic effects, we reduced the treatment frequency by a factor of two (Fig. 5f). The inflammation clearance observed on days 7 and 9 showed that the treatment remained effective, with significant inflammation clearance again. Subsequently, we reduced the treatment frequency to once daily. By day 11, the NO concentration continued to decrease markedly, further confirming that the inflammation had nearly completely cleared. This change was closely associated with the wound healing process; starting on day 5, scabbing began to form on the wound surface; by day 7, the wound started to close; and by day 11, the wound-closure rate exceeded 92%, nearly matching the healing progress observed in healthy mice (Figs. 5d, f, g, h, and S45). Furthermore, as the treatment duration was extended to day 20, a significant decrease in the NO current was still observed after wound healing, indicating that even after the wound had healed, the Pd-Ni_5_P_4_/DCEFS continued to effectively clear local inflammation (Figs. 5d, f, g, h, and S45). These results highlight the potential of the Pd-Ni_5_P_4_/DCEFS, through hydrogen generation, to accelerate diabetic mouse skin wound healing, particularly in terms of its significant therapeutic effects against inflammation and healing acceleration.

### Pd-Ni_5_P_4_/DCEFS Promotes Wound Healing by Modulating Macrophage Polarization and Tissue Remodeling

We conducted additional immunohistochemical (IHC) staining to evaluate the expression of the M1 marker (iNOS) and the M2 marker (Arg-1) within the wound tissues. The results revealed prominent positive expression of iNOS (indicated by brown areas) in the inflammation control group, reflecting a significant pro-inflammatory microenvironment; conversely, the Pd-Ni_5_P_4_/DCEFS-treated group exhibited a marked reduction in iNOS levels, accompanied by a substantial increase in Arg-1 positive expression. Furthermore, IHC sections showed that the tissue in the treatment group was more densely and orderly arranged (Fig. S46). These findings clearly demonstrate that Pd-Ni_5_P_4_/DCEFS effectively modulates macrophage polarization from the M1 to the M2 phenotype by downregulating iNOS and upregulating Arg-1, thereby promoting wound repair through the optimization of the immune microenvironment and providing direct experimental evidence for the biological efficacy of the system.

To further validate the downstream effects elicited by macrophage polarization, we conducted a histopathological assessment: H&E Staining: Histological sections revealed that the Pd-Ni_5_P_4_/DCEFS group exhibited a significant reduction in inflammatory cell infiltration, more mature granulation tissue formation, and a markedly accelerated re-epithelialization process. These observations are consistent with the trend of inflammatory resolution driven by the M1-to-M2 phenotypic transition. Masson’s Trichrome Staining: The results demonstrated denser and more highly organized collagen fiber deposition in the Pd-Ni_5_P_4_/DCEFS group. Given that M2 macrophages promote collagen synthesis through the secretion of growth factors (such as TGF-β), these findings provide further evidence supporting the successful modulation of macrophage function (Fig. 5i).

### Comprehensive Biosafety and Biocompatibility Evaluation of the Pd-Ni_5_P_4_/DCEFS System

While the therapeutic efficacy of this H_2_-driven approach is evident, ensuring the physiological safety of the electrochemical intervention is equally paramount for clinical application. To address the question of whether Pd-Ni_5_P_4_ nanoparticles might penetrate the wound bed, we have conducted a rigorous evaluation from the dual perspectives of physical barrier integrity and chemical dissolution: Within the system design, the Pd-Ni_5_P_4_ is integrated inside the DCEFS system. Even in the event of minimal macroscopic detachment, the outer microneedle array serves as an effective physical barrier, preventing large catalyst particles from directly contacting or infiltrating the wound tissue. To address the potential risk of ionic dissolution, we simulated clinical treatment regimen. During an 11-day simulated treatment test consisting of three sessions per day, ICP-MS results indicated that even by the final day, the concentration of Ni ions in the electrolyte remained as low as approximately 5.0 nM (Fig. S47). Furthermore, we performed stability test by maintaining a constant potential of − 0.25 V for 72 h. The current showed no significant degradation during this period. Subsequent ICP-MS analysis of the post-operational electrolyte confirmed that the Ni content remained consistently below 5.0 nM (Fig. S48). Literature studies [76–78] indicate that the threshold concentrations of nickel and palladium ions required to induce toxic reactions in mice are significantly higher than the 5.0 nM level detected in our system (Fig. S48). Consequently, the Pd-Ni_5_P_4_/DCEFS exhibits exceptional structural integrity under physiological conditions. The resulting ionic leaching is negligible, posing a minimal and inconsequential risk to biological systems.

We conducted comprehensive hematological and biochemical analyses on normal mice (Normal) and treated diabetic mice (Treatment Group), as presented in Tables S4-S7. The results demonstrate that throughout the treatment process, key hematological parameters of the experimental mice-including white blood cell (WBC) count, red blood cell (RBC) count, hemoglobin (HGB), and platelet (PLT) count-remained within reasonable physiological ranges, with no abnormal deviations attributable to the material. In the biochemical evaluation of liver function (ALT, AST) and renal function (BUN, CREA), the treatment group exhibited no statistically significant pathological damage compared to the control group (Tables S4-S7). Although the diabetic group displayed expected pathological variations in certain metabolic indicators, the overall consistency of these biochemical parameters indicates that the material induced no detectable acute systemic toxicity or severe inflammatory response during the experimental period. These findings confirm that the nanomaterial possesses excellent biosafety for in vivo applications. We harvested the primary organs (heart, liver, spleen, lungs, and kidneys) from mice in each group after 11 days of treatment for hematoxylin and eosin (H&E) staining (Fig. S49). The organ morphology in the Pd-Ni_5_P_4_/DCEFS-treated group remained normal, with no evidence of significant tissue necrosis, cellular degeneration, or pathological inflammatory infiltration. These results provide preliminary evidence of the material's excellent biocompatibility.

The potential applied during the treatment process was only − 0.25 V, an energy input significantly below the threshold for biological tissue damage. From an electrochemical standpoint, this potential is substantially lower than the theoretical electrolysis voltage of water (1.23 V), effectively averting the risk of chemical burns caused by the generation of gas bubbles or the creation of localized extreme pH (strong acidic or alkaline) environments. Furthermore, the extremely low current density ensures that Joule heating during the treatment is negligible, thereby precluding the possibility of thermal injury and confirming the mild and non-invasive nature of the therapeutic intervention. To further verify the practical in vivo safety of this potential, a detailed histopathological assessment of the treated area was conducted. As shown in the Hematoxylin and Eosin (H&E) staining results (Figs. 5i and S50), the tissue morphology following the − 0.25 V treatment remained indistinguishable from the control group, with the epidermis, dermis, and adnexal structures remaining intact. No cell necrosis, morphological degeneration, or significant inflammatory cell infiltration was detected during the observation. These findings provide direct evidence at the cellular and tissue levels that the weak electrical stimulation protocol exhibits excellent biocompatibility and does not induce perceptible pathological damage. The Pd-Ni_5_P_4_/DCEFS demonstrated excellent dynamic therapeutic effects in clearing inflammation in the wound area and promoting skin wound healing in diabetic mice, providing a potentially effective strategy for treating diabetic wounds.

## Conclusions

In this study, we designed an innovative dual-channel electrocatalytic flexible system for the dynamic treatment of diabetic wounds. This system not only enables digital monitoring of the inflammation clearance process but also accelerates wound healing through hydrogen gas in a cyclic dynamic treatment. Through real-time monitoring and precise control, the system can accurately track the inflammation clearance process and analyze the speed of wound healing. The experimental results showed that by day 5 of treatment, the system detected a significant reduction in inflammation and accelerated the healing process. By day 11, inflammation was effectively cleared, and wound healing was nearly complete. This dual-channel flexible electrocatalytic system offers a novel approach for the design of dynamic therapeutic systems and provides robust technical support for the implementation of personalized treatment. We believe that this system design opens new avenues for building highly sensitive, efficient, and stable dynamic treatment platforms, advancing the precise assessment and treatment of wound healing in complex biological systems, with significant clinical application potential.

## Supplementary Information

Below is the link to the electronic supplementary material.

Supplementary file1 (DOCX 11918 KB)
