# Supplementary material for: Closed-Loop Synergistic Nitric Oxide/Hydrogen Delivery with Feedback Control for Diabetic Wound Healing
Source: Nanomicro Lett. 2026 May 26;18:385. doi: 10.1007/s40820-026-02237-1 (PMC13201802; doi:10.1007/s40820-026-02237-1)
Supplement: Supplementary file 1 — Supplementary file1 (DOCX 11918 KB) [file 40820_2026_2237_MOESM1_ESM.docx]

Supporting Informations for

**Closed-Loop Synergistic Nitric Oxide/Hydrogen Delivery with Feedback Control for Diabetic Wound Healing**

Pengfei Wen^1^†*, Pan Luo^2^†, Fuqiang Gao^3,4^†, Mingyi Yang^1^†*, Junyou Li^5^, Zhi Yang^1^*

^1^Department of Joint Surgery, Honghui Hospital, Xi’an Jiaotong University, Xi’an 710054, P. R. China

^2^Department of Plastic Surgery, Beijing Chaoyang Hospital Affiliated to Capital Medical University, Beijing 100020, P. R. China

^3^Center for Hip Preservation, Osteonecrosis and Developmental Dysplasia of the Hip, China-Japan Friendship Hospital, Beijing 100029, P. R. China

^4^Department of Orthopedics, China-Japan Friendship Hospital, Beijing 100029, P. R. China

^5^School of Mechanical Engineering, Sungkyunkwan University, Suwon 16419, South Korea

† Pengfei Wen, Pan Luo, Fuqiang Gao, and Mingyi Yang contributed equally to this work.

*Corresponding authors. E-mail: [wenpengfei@pku.edu.cn](mailto:wenpengfei@pku.edu.cn) (Pengfei Wen); [ymy25808@stu.xjtu.edu.cn](mailto:ymy25808@stu.xjtu.edu.cn) (Mingyi yang); [yangzhi@xiyi.edu.cn](mailto:yangzhi@xiyi.edu.cn) (Zhi Yang)

**Supplementary Figures and Tables**


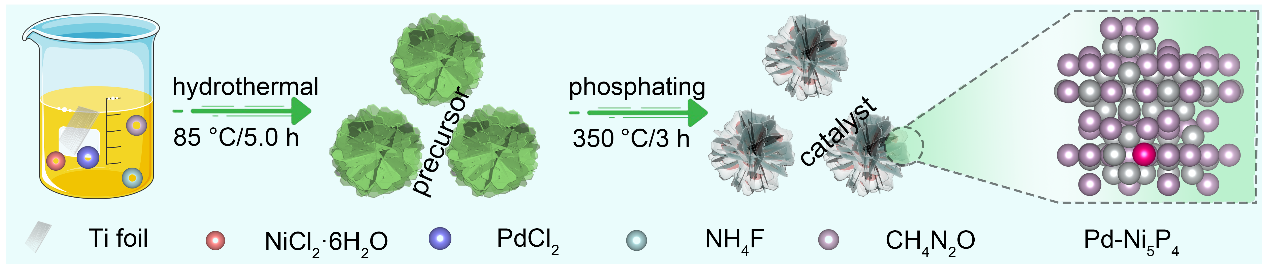


**Fig. S1** Schematic of the synthesis of Pd-Ni_5_P_4_/Ti.


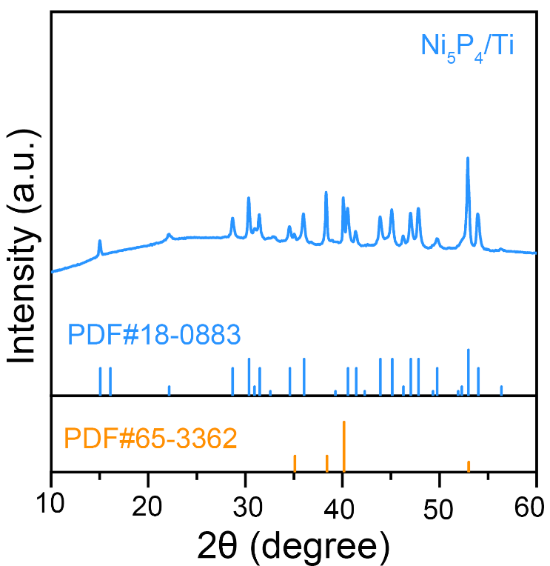


**Fig. S2** XRD patterns of Ni_5_P_4_ supported on Ti foil.


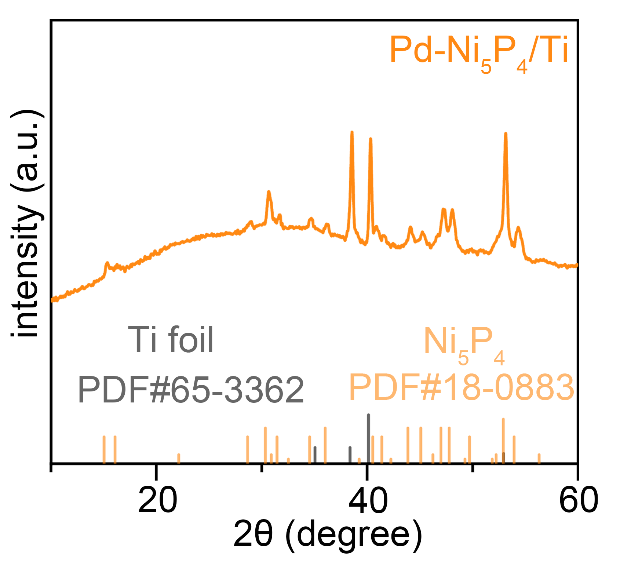


**Fig. S3** XRD patterns of Pd-Ni_5_P_4_ grown on Ti foil (scan rate: 5.0°/min).


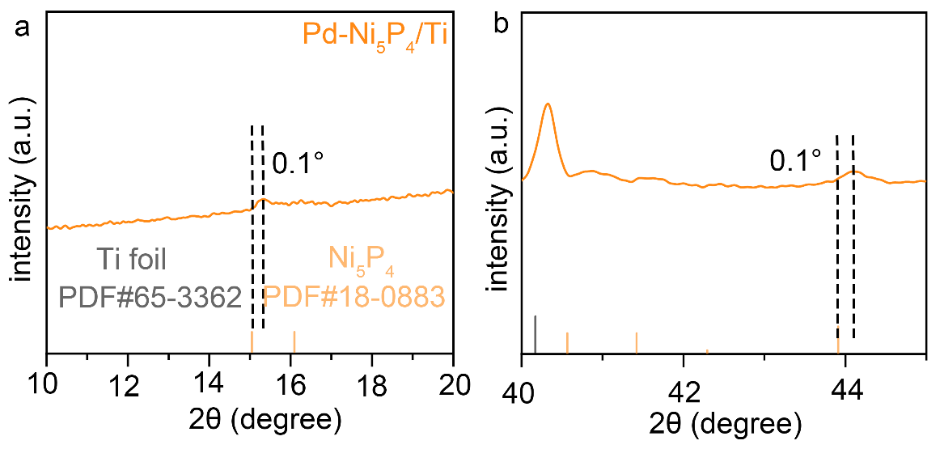


**Fig. S4** XRD patterns of Pd-Ni_5_P_4_ supported on Ti foil. a 10-20°. b 40-45°.


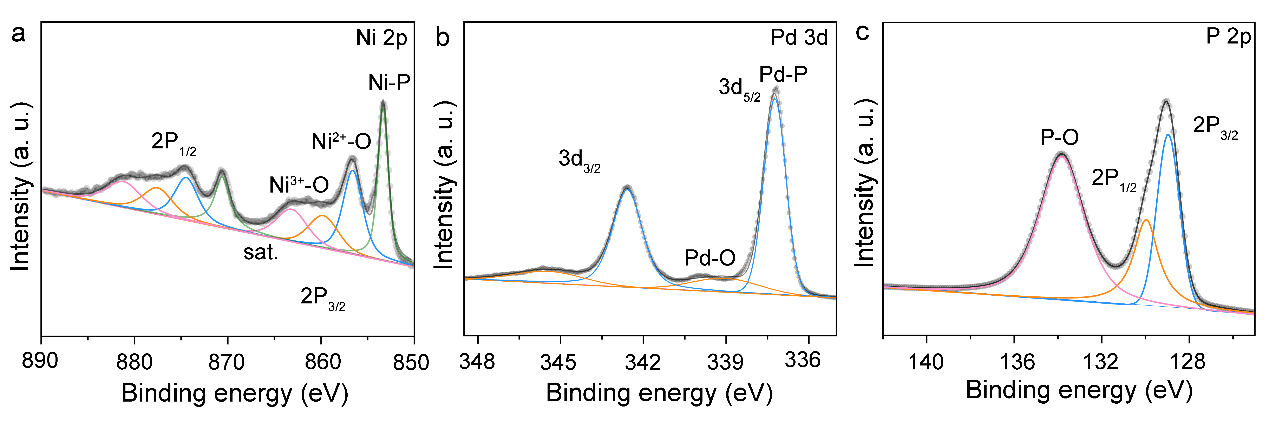


**Fig. S5 a** Ni 2p, **b** Pd 3d and **c** P 2p XPS spectra of Pd-Ni_5_P_4_ catalysts.


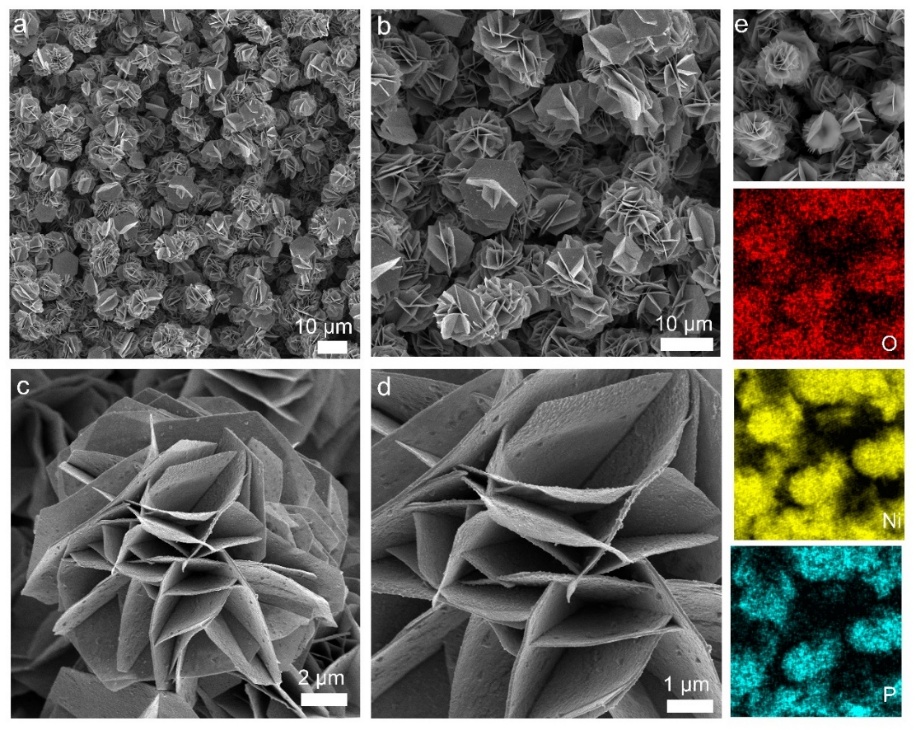


**Fig. S6** Characterizations of the morphology and microstructure of Ni_5_P_4_/Ti. SEM images: **a** scale bar: 10.0 μm. **b** scale bar: 10.0 μm. **c** scale bar: 2.0 μm. **d** scale bar: 2.0 μm. **e** EDS mapping of a Ni_5_P_4_/Ti.


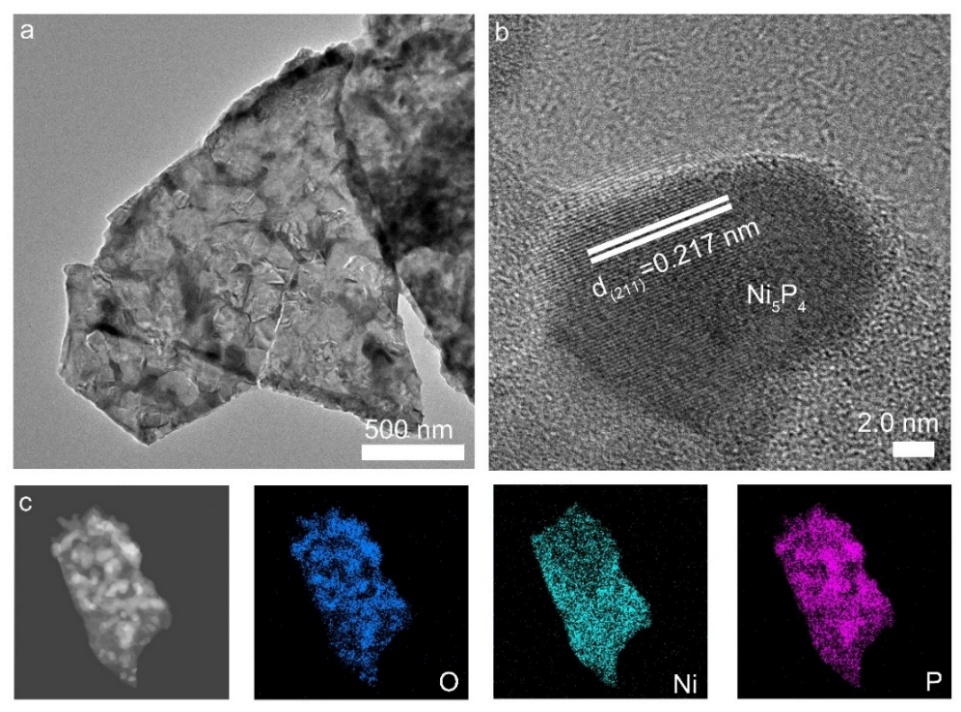


**Fig. S7** Ni_5_P_4_/Ti: **a** TEM image. **b** HRTEM image. **c** TEM mapping of Ni_5_P_4_.


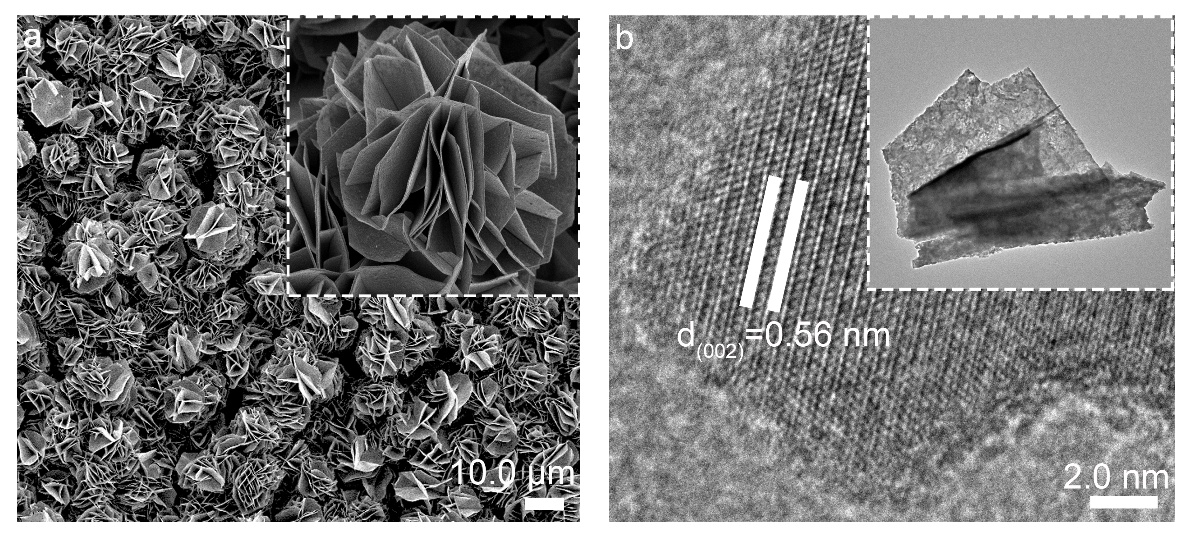


**Fig. S8** Pd-Ni_5_P_4_/Ti: **a** SEM images. Insert: enlarged SEM image. **b** HRTEM image. Insert: TEM image of nanosheet structure.


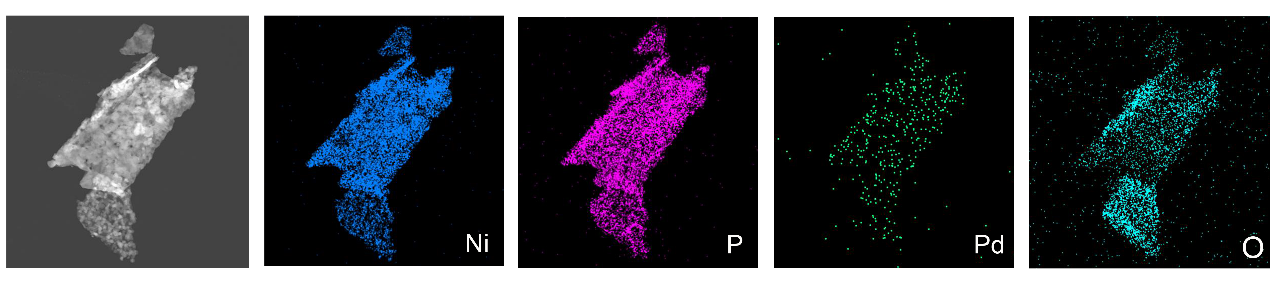


**Fig. S9** TEM mapping of Pd-Ni_5_P_4_ nanosheet.


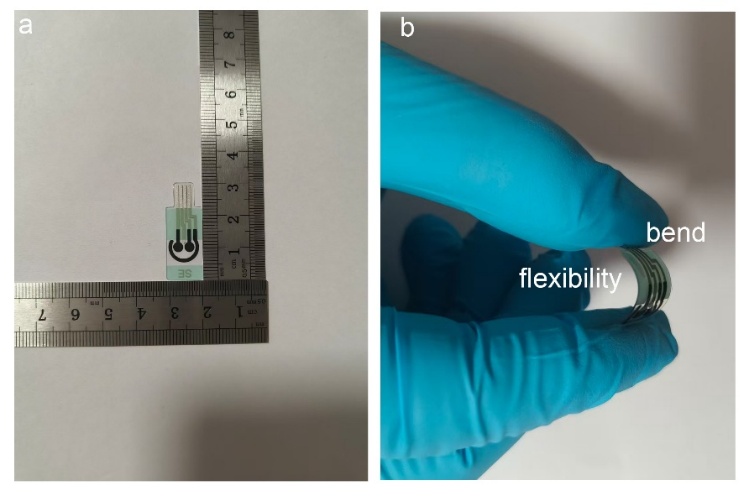


**Fig. S10** **a** Size of the dual-channel electrodes. **b** Digital photograph of the Pd-Ni_5_P_4_/DCEF with bending.


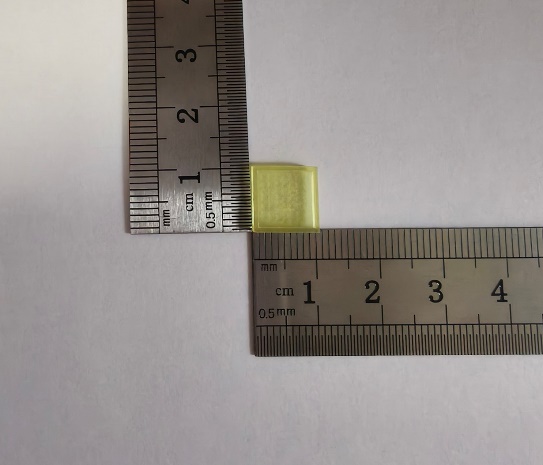


**Fig. S11** The size diagram of the microneedle.


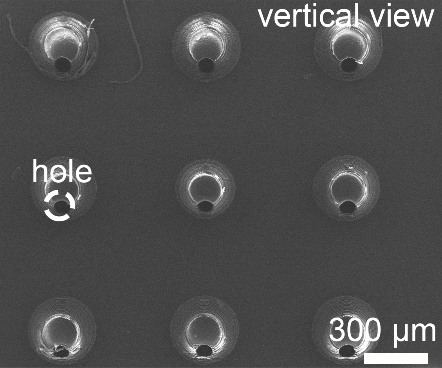


**Fig. S12** SEM image of the pores in the microneedle array.


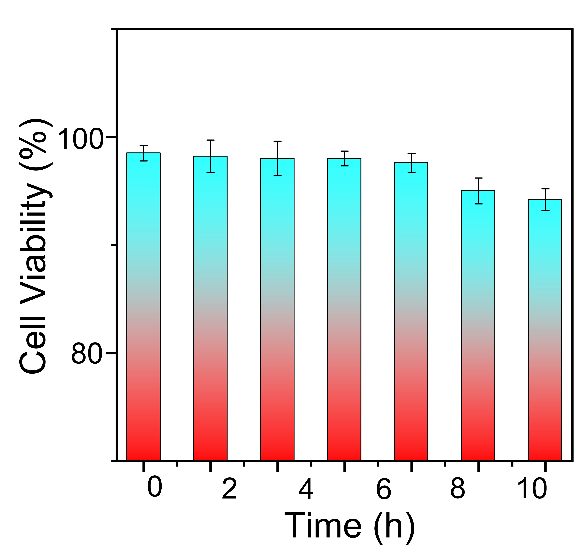


**Fig. S13** CCK-8 assay of RAW 264.7 cells co-incubated with microneedle array for different time periods.


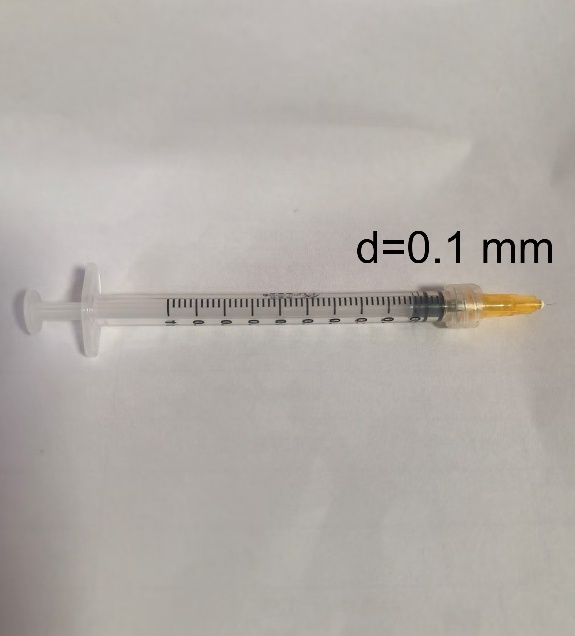


**Fig. S14** Electrolyte charging device.


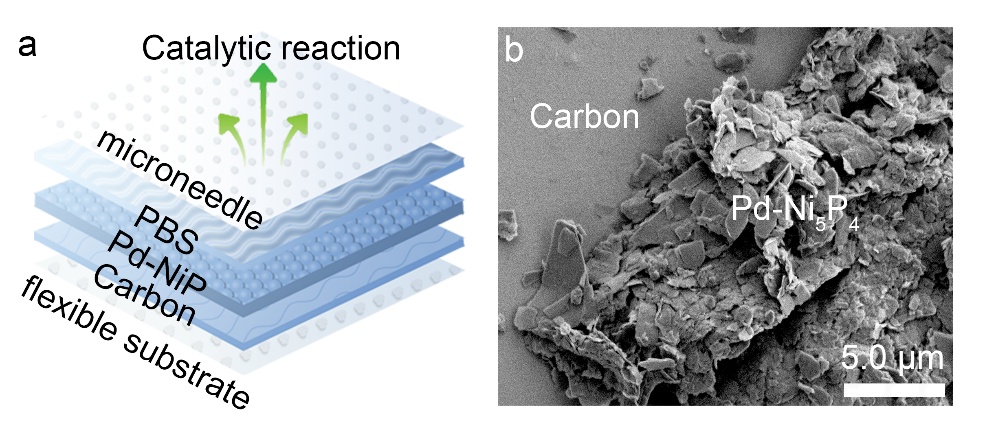


**Fig. S15 a** Schematic diagram of the cross-section of the DCEFS. **b** SEM spectrum of CB.


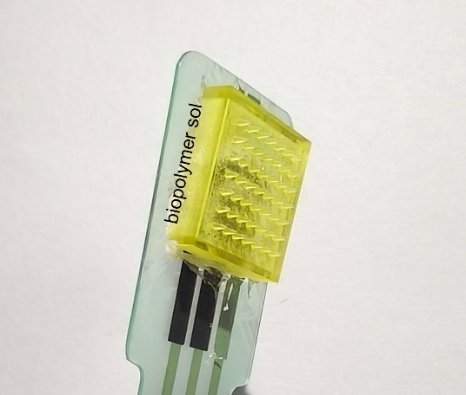


**Fig. S16** Biopolymer sol effectively combines microneedle arrays with DCFE.


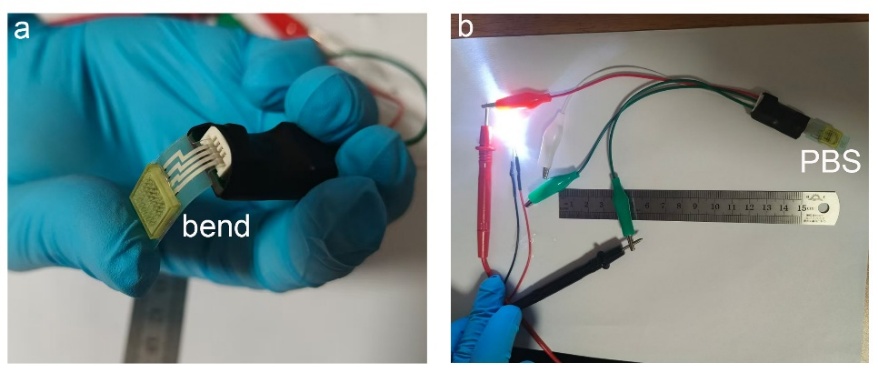


**Fig. S17 a** Image of the DCEFS after bending deformation with PBS solution loaded. **b** Image showing the brightness of the LED light after applying power to the DCEFS with PBS solution loaded.


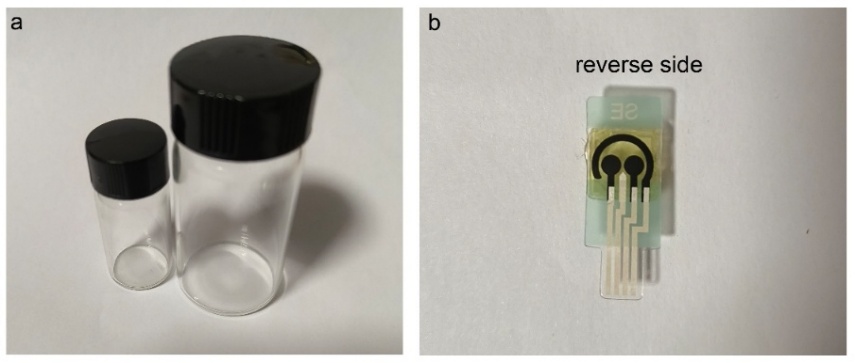


**Fig. S18 a** Different sizes of bottles. **b** Opposite of the system.


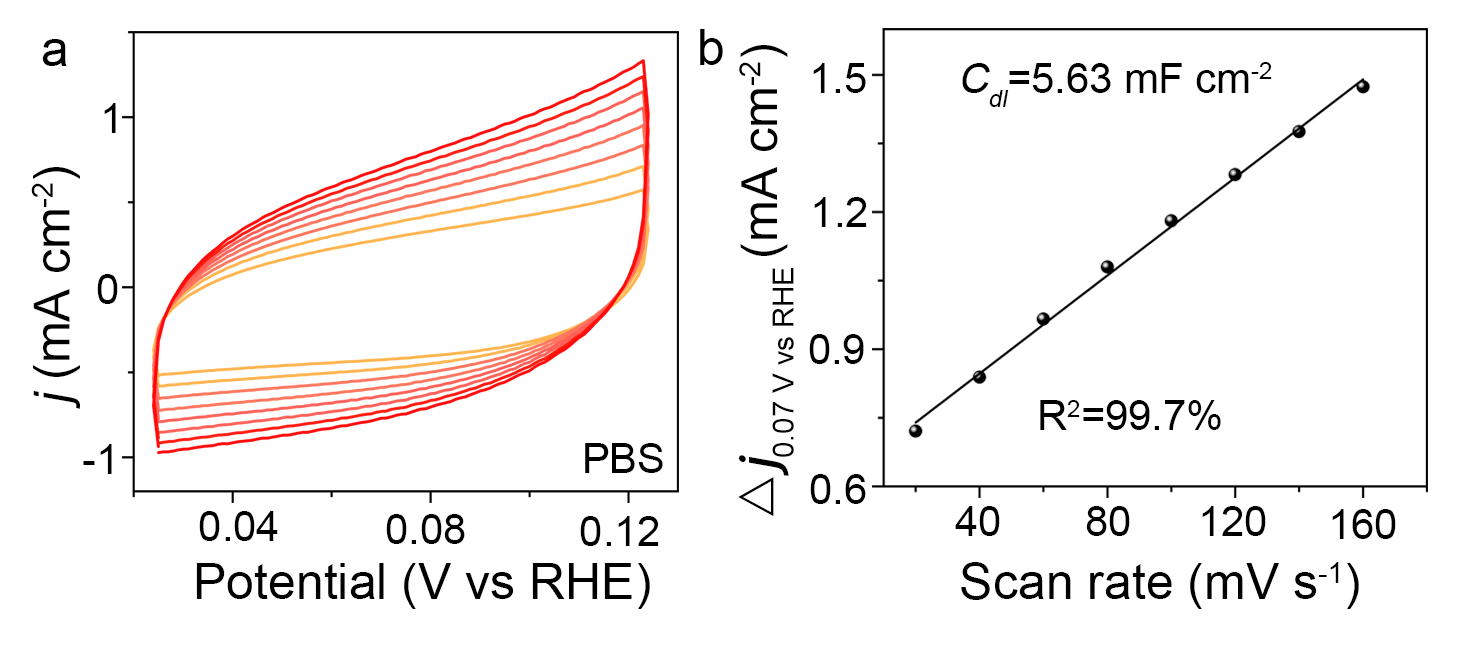


**Fig. S19 a** Cyclic voltammetry (CV) curves of Pd-Ni_5_P_4_ recorded at various scan rates ranging from 20 to 160 mV s^-1^. **b** Capacitive current density at 0.07 V as a function of the scan rate.


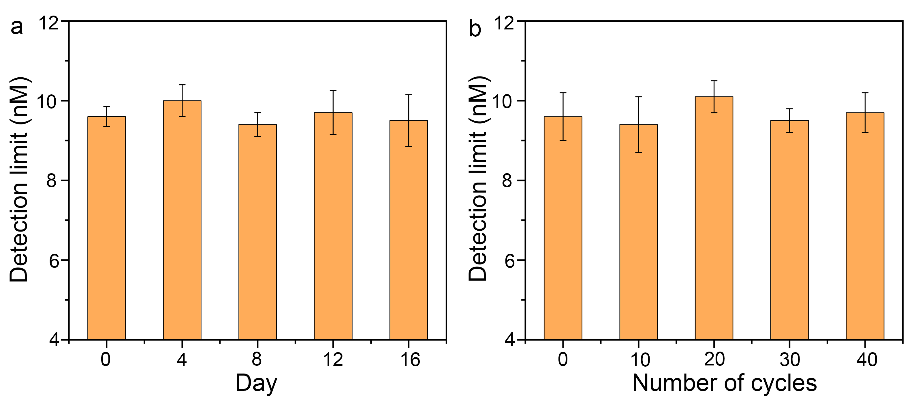


**Fig. S20 a** Stability testing of the sensor over time dimension. **b** Stability testing of the sensor through multiple cyclic tests.


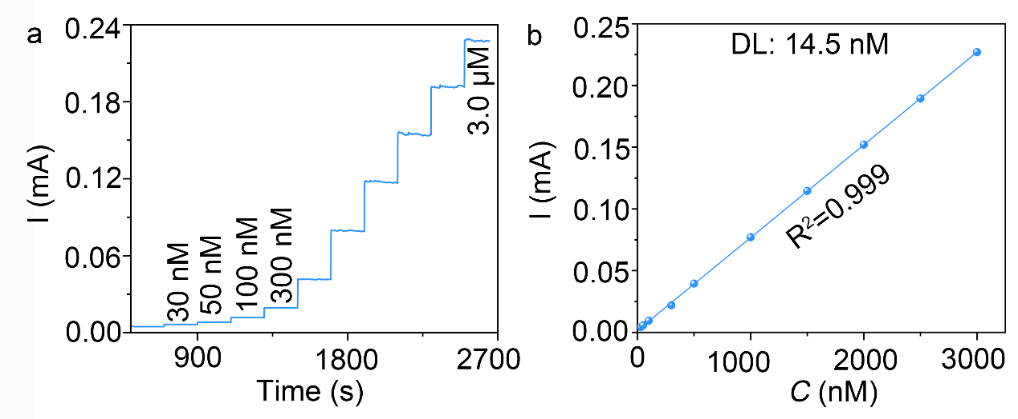


**Fig. S21 a** Amperometric i-t response of the Pd-Ni_5_P_4_/DCEFS sensor to varying concentrations of NO in a complex medium (culture medium + blood) at an applied bias of 0.75 V (detection range: 30 nM to 3.0 μM). **b** Calibration curve illustrating the linear relationship between the response current and NO concentration.


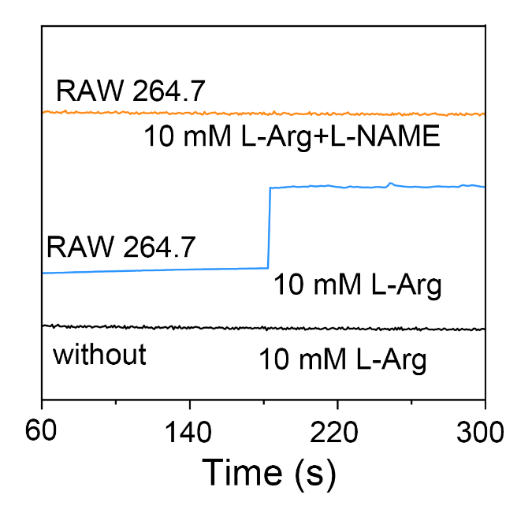


**Fig. S22** Monitoring NO release from without cells after the addition of 10 mM L-Arg (black curve). Curves represent the current responses under stimulation of 10 mM L-Arg and L-NAME (orange curve) and 10 mM L-Arg (blue curve) with HUVECs.


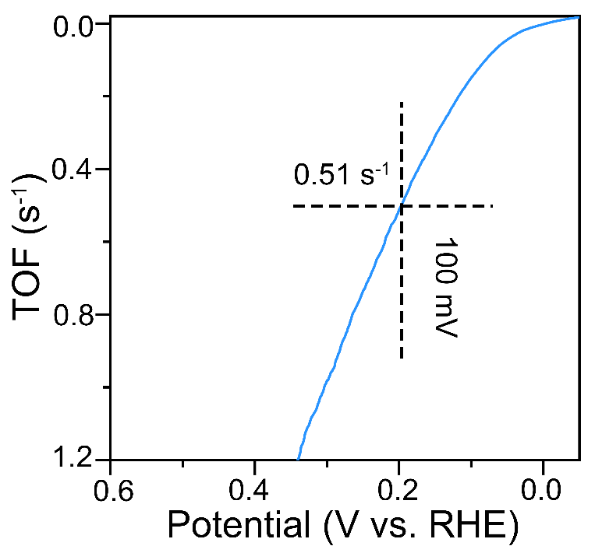


**Fig. S23** TOF values of Pd-Ni_5_P_4_/Ti.

The TOFs [s^−1^] were calculated by Equation: TOF=I/2nF where I is current [A] during the linear sweep measurement after 10 modification cycles, F is the Faraday constant [C mol^−1^], and n is the number of active sites [mol] after 10 modification cycles. A factor of 1/2 is arrived at by taking into account that two electrons are required to form one hydrogen molecule from two protons.


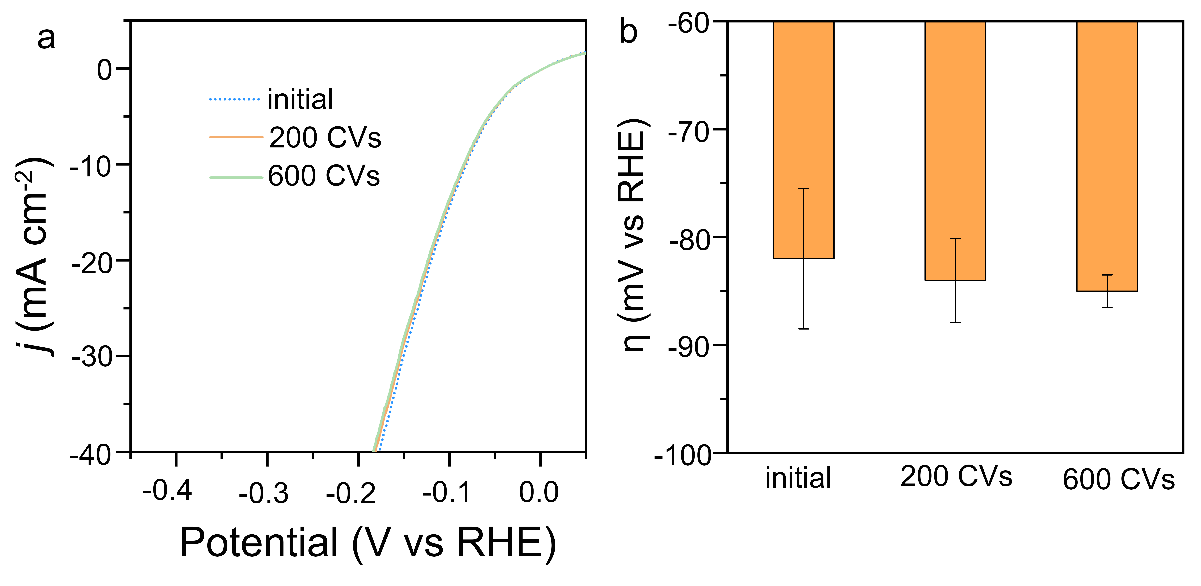


**Fig. S24 a** LSV curves of CoP before and after 200 and 600 cycles of a durability test with potential between 0.0 and -0.8 V (versus RHE) and sweep rate of 100 mV s^-1^. At the end of the cycling test, the electrode was used for polarization curves investigation at a sweep rate of 5 mV s^-1^. **b** The relationship graph between cycle number and overpotential (corresponding to -10.0 mA cm^-2^).


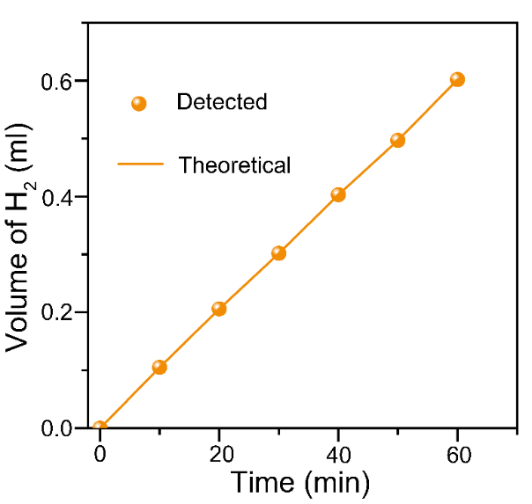


**Fig. S25** Amount of H_2_ calculated (solid) and measured (sphere) versus time for Pd-Ni_5_P_4_/Ti at pH=7.4 under an overpotential of -250.0 mV for 60.0 min.


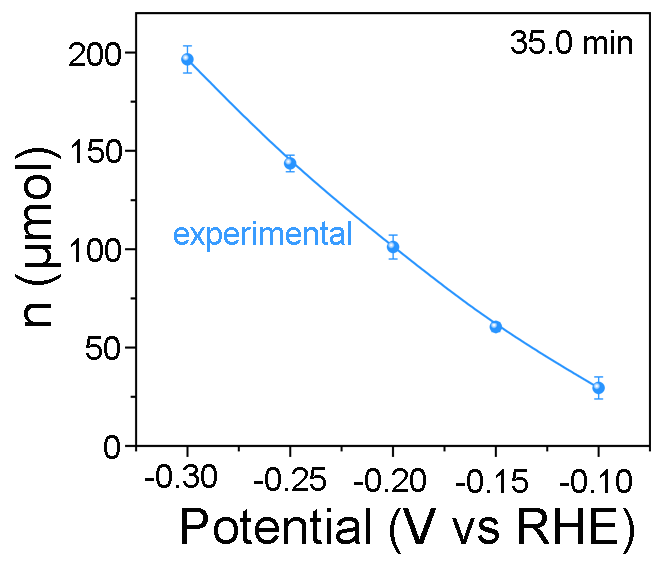


**Fig. S26** The intrinsic dose-response relationship between applied voltage and hydrogen yield established based on Faraday’s Law of Electrolysis, along with the actual hydrogen production measured via the water displacement method.


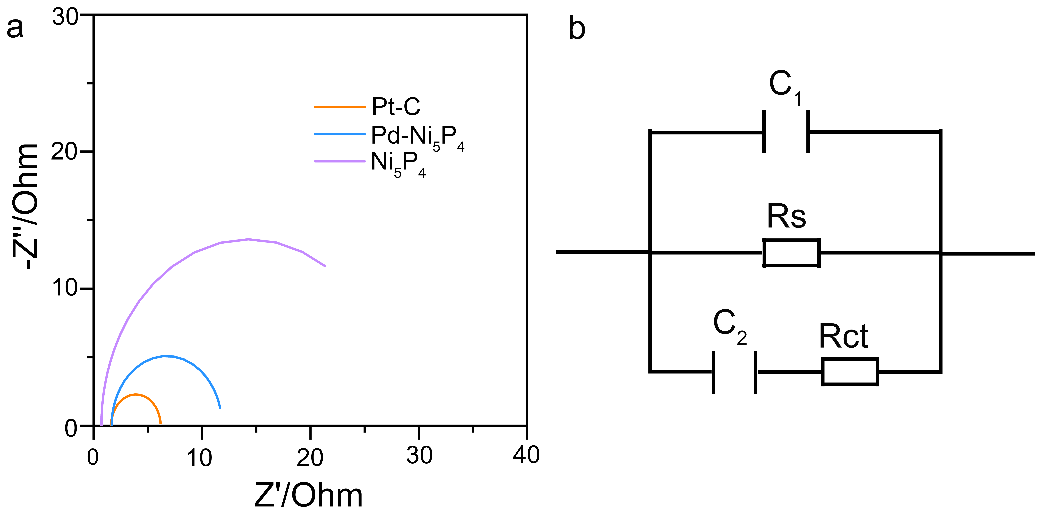


**Fig. S27 a** Nyquist plots of the different samples with an applied bias of -0.4 V (vs RHE). **b** Simulate circuit diagram.


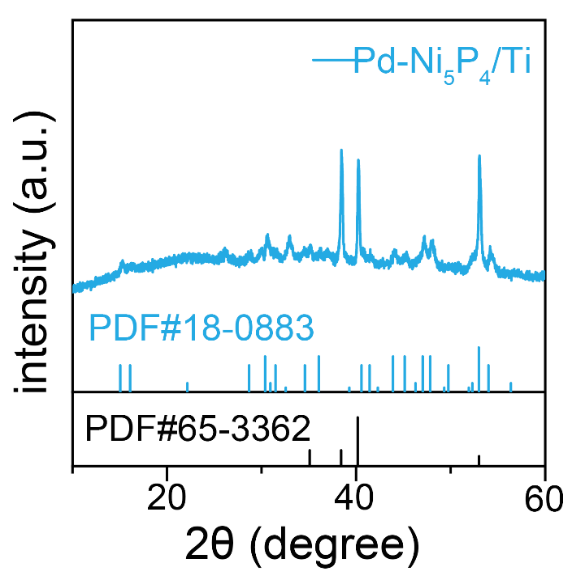


**Fig. S28** XRD patterns of Pd-Ni_5_P_4_/Ti after HER.


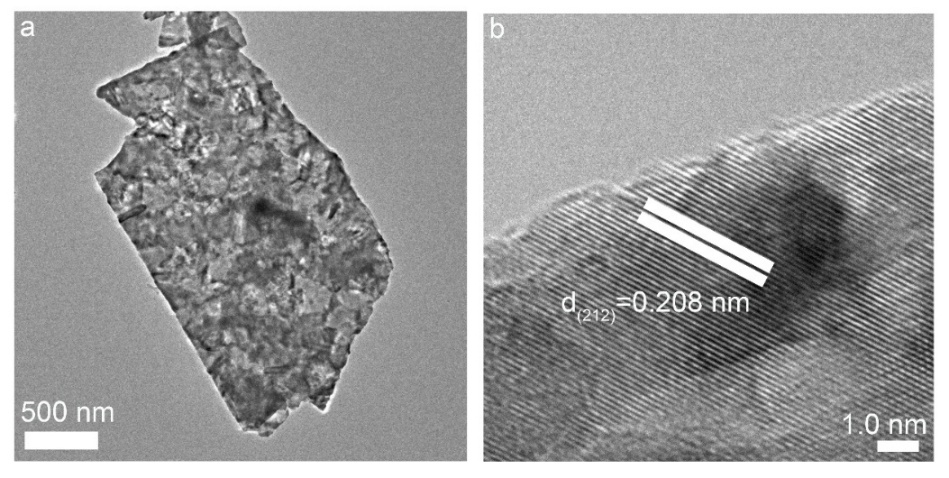


**Fig. S29** After HER: **a** TEM image of Pd-Ni_5_P_4_/Ti microspheres. **b** HRTEM image (scale bar: 1.0 nm).


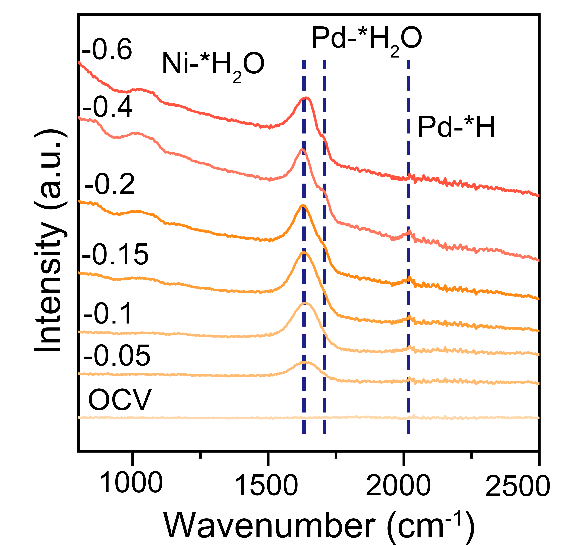


**Fig. S30** In situ Raman spectra of Pd-Ni_5_P_4_/Ti recorded in PBS solution (pH=7.4) at OCV from -0.6 to -0.05 V vs. RHE.


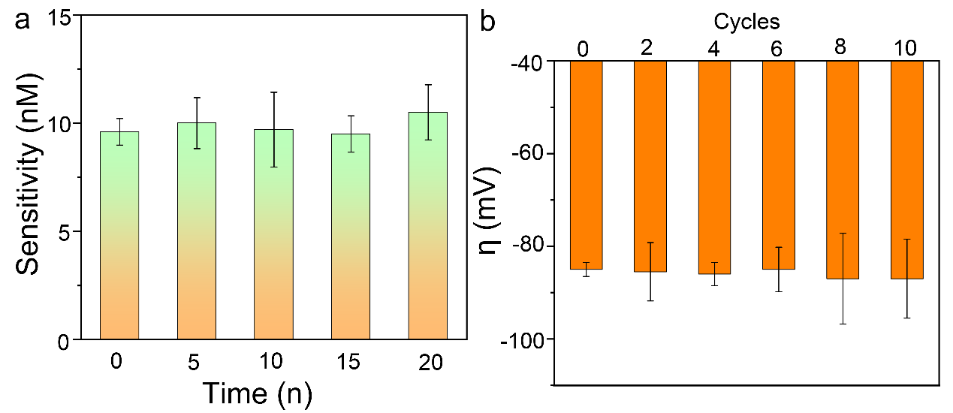


**Fig. S31** Multiple cyclic tests using the adapter were performed to verify its: **a** Repeatability of the electrochemical sensor's detection limit. **b** Stability of the hydrogen evolution performance.


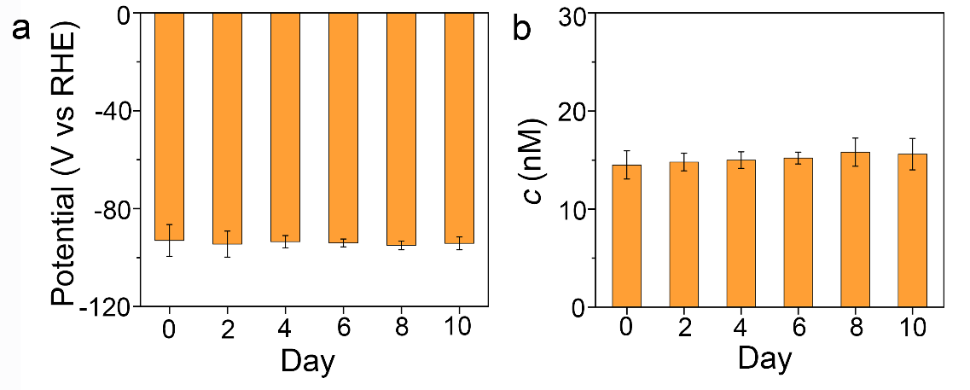


**Fig. S32** Stability assessment of Pd-Ni_5_P_4_/DCEFS in biological culture media. **a** Trend of the hydrogen evolution overpotential at a current density of 10 mA cm^-2^ over a 0-10 days immersion period. **b** Evolution of the sensor's limit of detection (LOD) for NO during the 0-10 days immersion. All experiments were conducted in a culture medium environment at 37 °C; error bars represent the standard deviation of three independent measurements (n = 3).


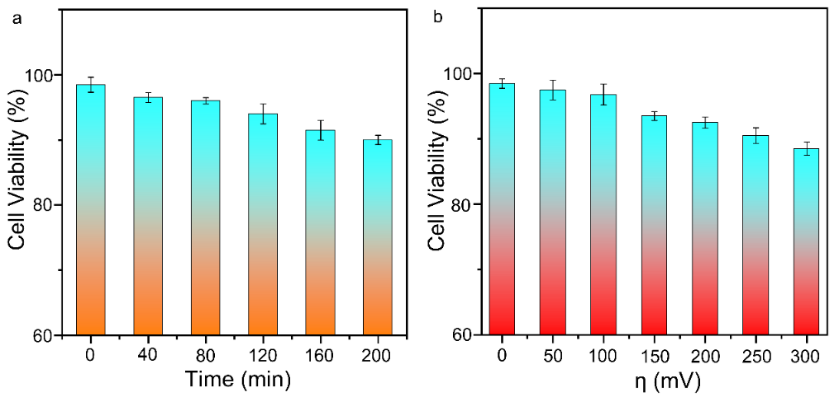


**Fig. S33 a** Place the Pd-Ni_5_P_4_/DCEFS in PBS culture solution, set the voltage to open-circuit voltage, and study the relationship between cell viability and time. **b** Use the Pd-Ni_5_P_4_/DCEFS to generate hydrogen for 10.0 minutes under different voltages, and plot the relationship between cell viability and different voltages. The experiments were repeated three times and the data were shown as mean (± S.D.).


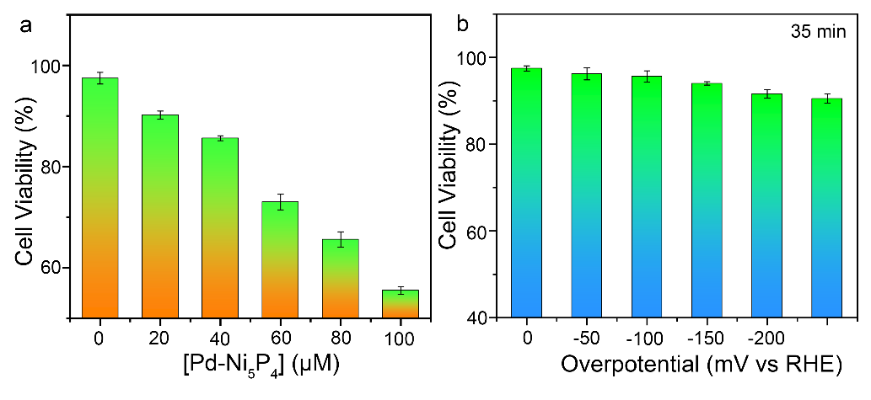


**Fig. S34 a** MTT assay of Raw 264.7 cells with Pd-Ni_5_P_4_ at different concentration (0-100.0 μM). **b** The evaluation of cell viability of Pd-Ni_5_P_4_/DCEFS at different voltages. The experiments were repeated three times and the data were shown as mean (± S.D.).


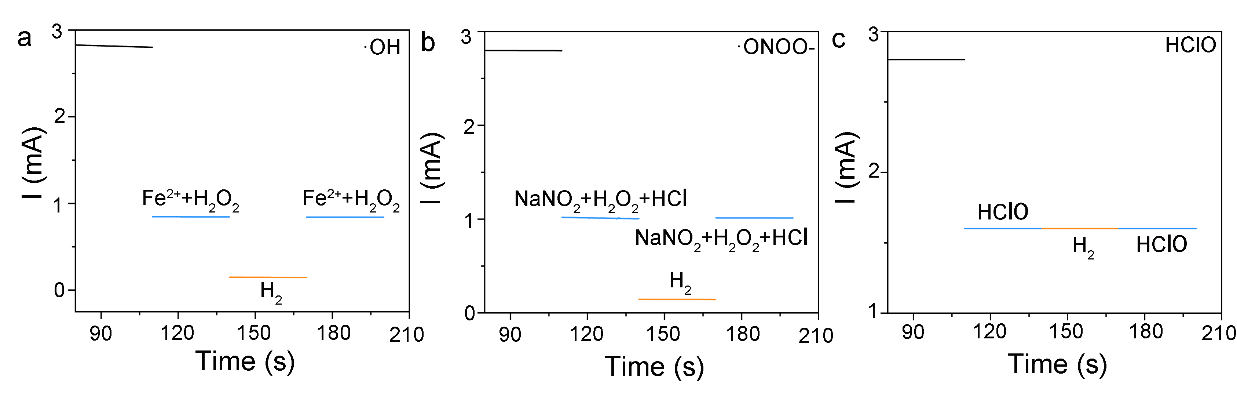


**Fig. S35** Selective scavenging capacity of H_2_ toward different ROS species. **a–c** Representative chronoamperometric (I-t) curves obtained from the dual-channel electrode system in the presence of specific reactants: **a** ∙OH (generated from Fe^2+^+H_2_O_2_), **b** ONOO^-^ (generated from NaNO_2_ + H_2_O_2_ + HCl), and c HClO. The black lines represent the hydrogen evolution current at Channel A. At Channel B (detection electrode), the introduction of ROS precursors leads to the establishment of new steady-state current levels (blue lines). Subsequent activation of H_2_ production (orange lines) results in a significant reduction of the current signals in the **a** ∙OH and **b** ONOO^-^ systems, whereas the impact on the **c** HClO system is negligible. These results demonstrate the high selectivity of H_2_ toward highly reactive oxygen radicals.


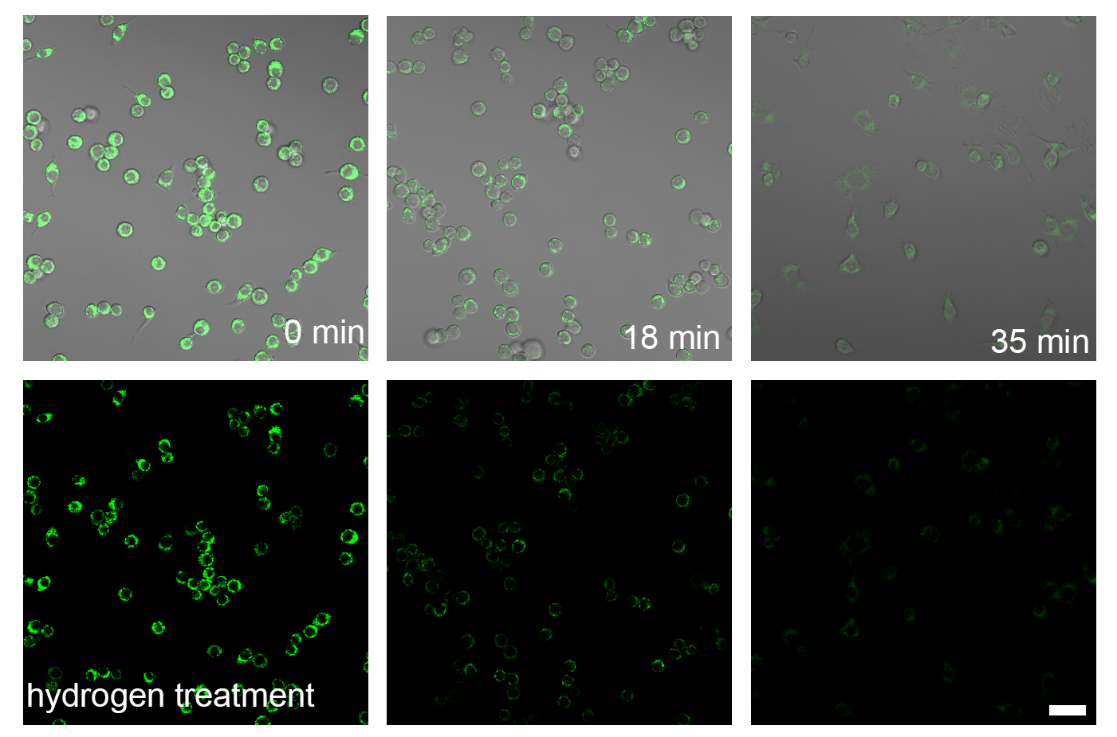


**Fig. S36** During hydrogen treatment, the release of hydrogen at different time (0, 18 and 35 min) points affects the degree of inflammation resolution and cell differentiation. Scale bars: 50 μm.


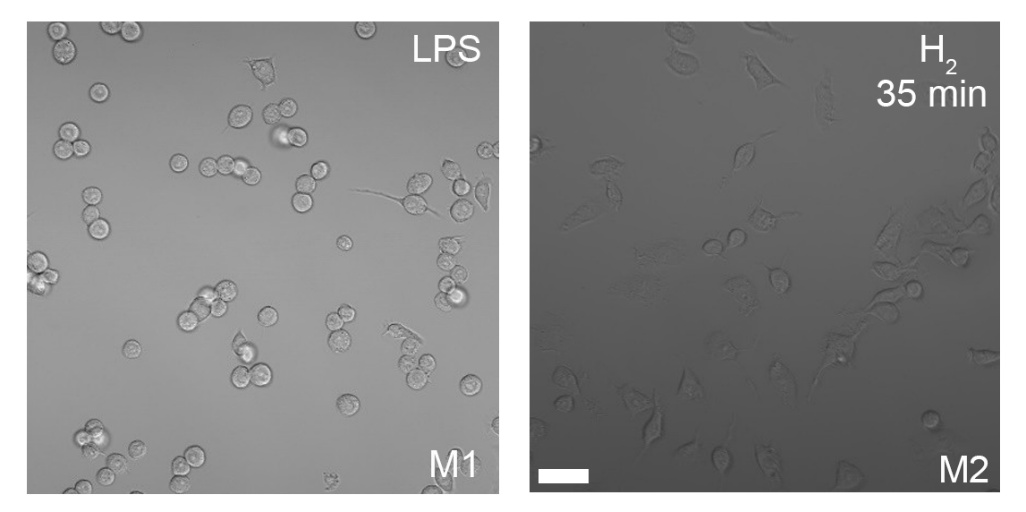


**Fig. S37** Cell differentiation status during a LPS and b hydrogen treatment. Scale bars: 50 μm.


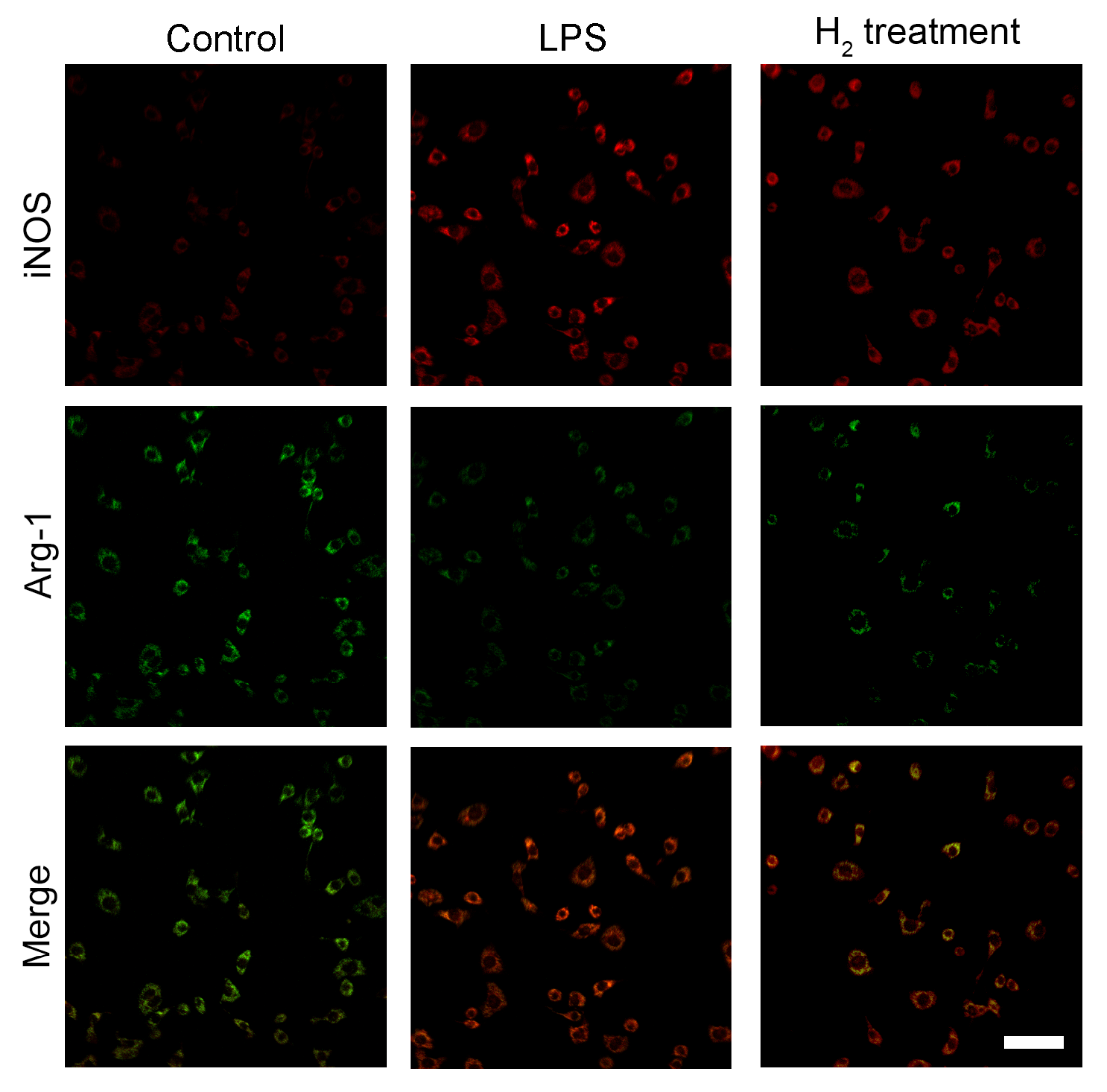


**Fig. S38** Representative immunofluorescence (IF) images of iNOS (red, M1 marker) and Arg-1 (green, M2 marker) in macrophages across different groups (Control, LPS, and H2 treatment). The results indicate that H2 treatment effectively suppresses the LPS-induced upregulation of iNOS while promoting the expression of Arg-1. Merged images show the co-staining of the two markers. Scale bars: 50 μm.


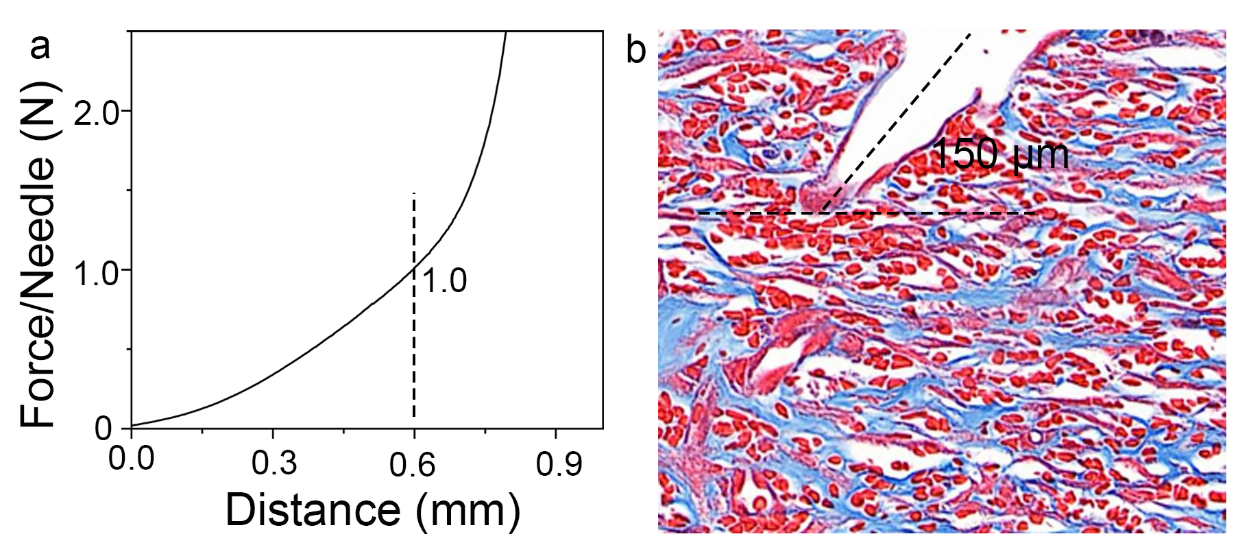


**Fig. S39 a** Force-displacement curves of the microneedle under different applied forces. **b** H&E staining of mouse skin after administration of microneedle patch.


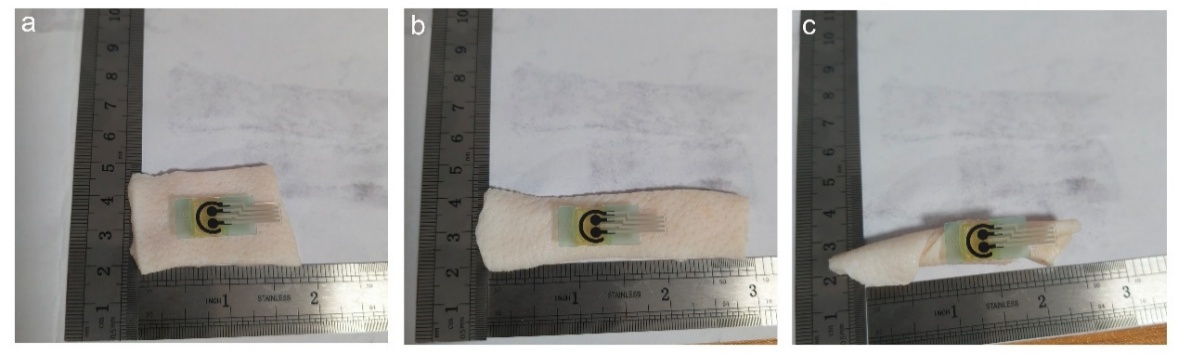


**Fig. S40** Adhesive ability of microneedle patch in dry air.


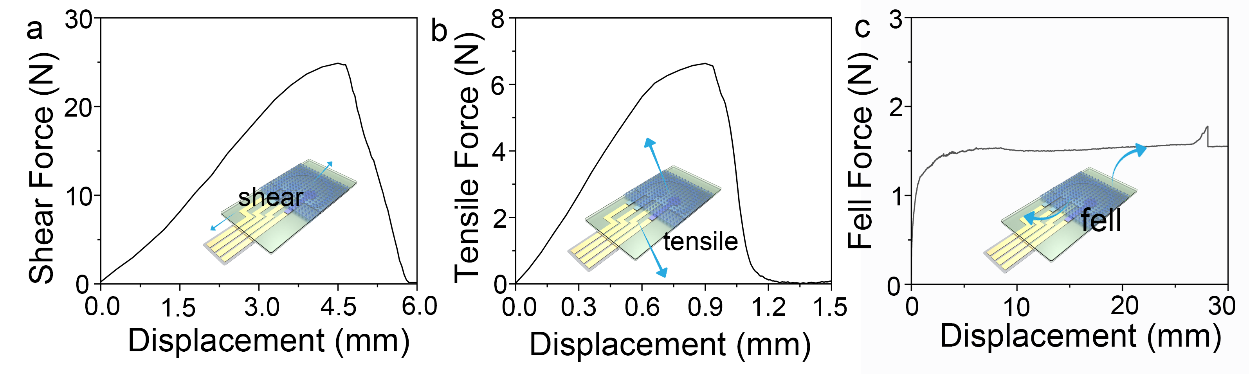


**Fig. S41** Measurement of the Adhesion Performance of Pd-Ni_5_P_4_/DCEF on Mouse Skin: **a** Schematic illustrations and corresponding force-displacement curves for the shear, **b** tensile, and c peeling processes.


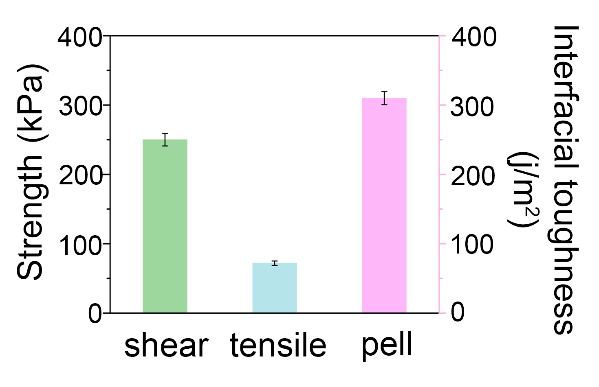


**Fig. S42** Data for shear strength, tensile strength, and interfacial toughness.


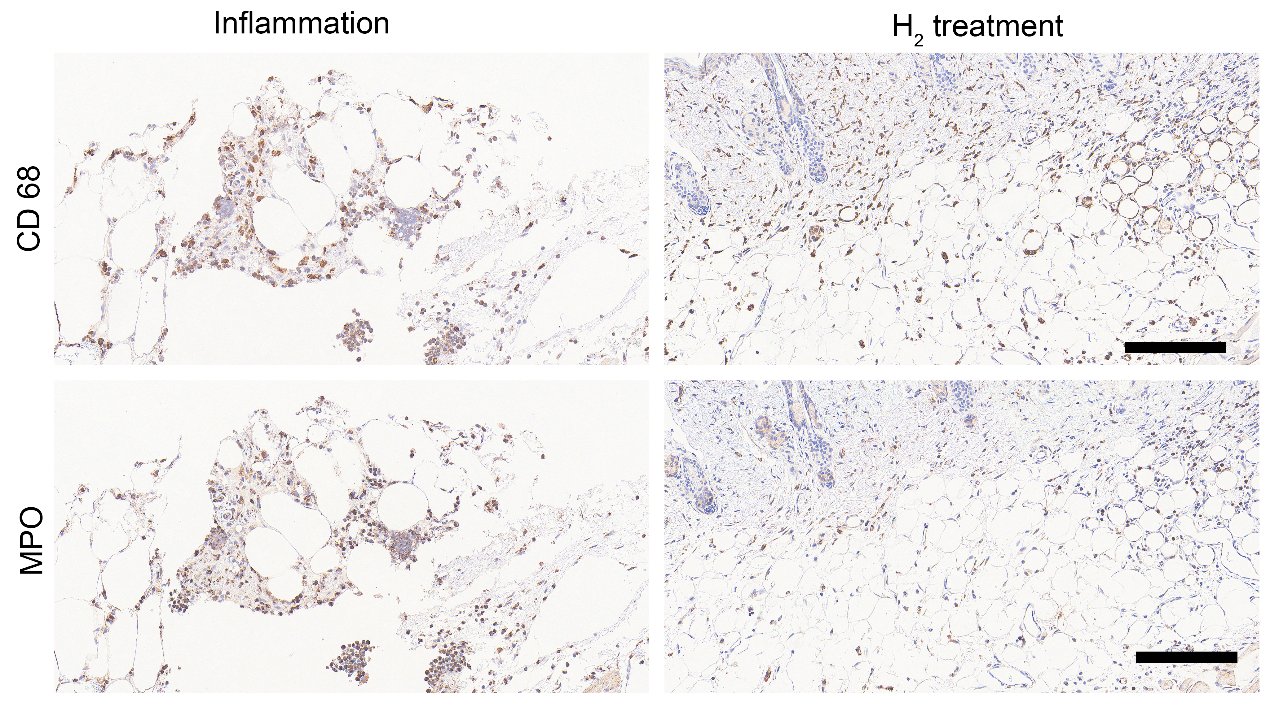


**Fig. S43** Representative immunohistochemical (IHC) images of CD68 and MPO in the wound tissues of diabetic mice. The images compare the levels of immune cell infiltration between the inflammation control group and the H_2_ treatment group. Scale bars: 100 μm.

The function of calibration curve:

$$y= y_{0}+\frac{a}{1+e^{\frac{x-x_{0}}{b}}}$$

a = 5.3406, b = 10.9955, x_0_ = 23.0711, y_0_ = 0.8102


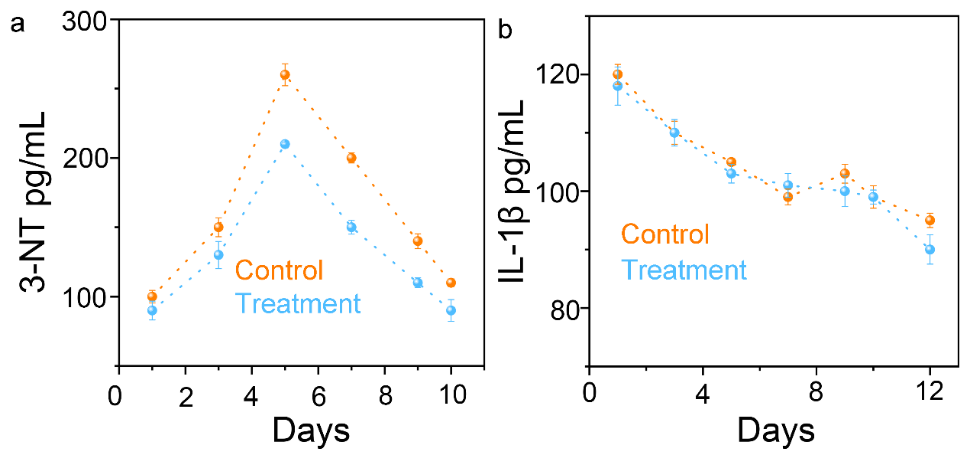


**Fig. S44** Examining the variation curves of inflammatory factors in the blood near the wound over time under conditions with and without hydrogen therapy. **a** Blood samples tests of 3-NT using ELISA kits at 1st, 3rd, 5th, 7th, 9th, and 11th day. **b** Blood samples tests of IL-1β using ELISA kits at 1st, 3rd, 5th, 7th, 9th, and 11th day. The error bar represents the standard deviation of three independent measurements (n = 4, mean ± SD).


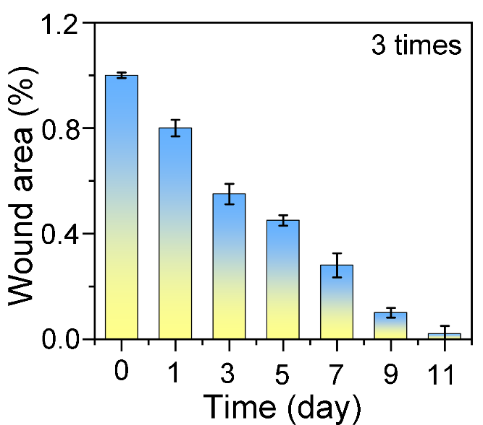


**Fig. S45** The wound healing progress at different time points (days) is assessed through the dynamic treatment system, three times a day.


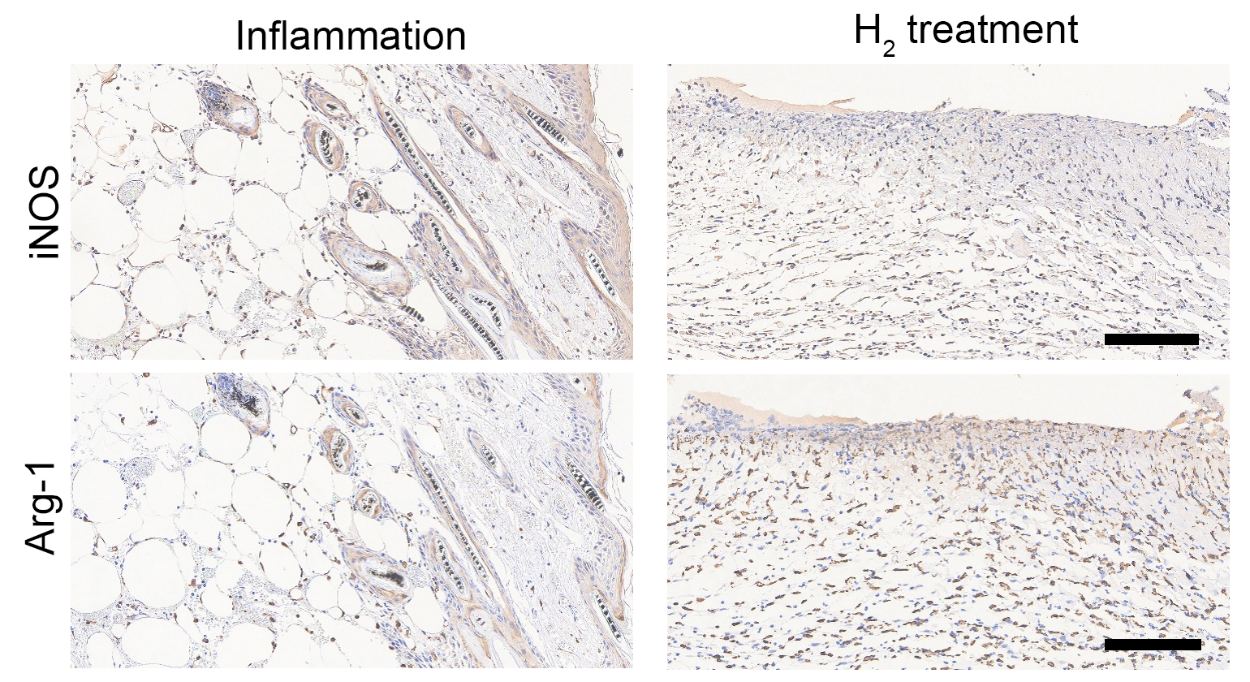


**Fig. S46** H_2_ treatment modulates macrophage polarization from M1 to M2 in diabetic wound tissues. Representative immunohistochemical images of wound tissue sections stained for iNOS (pro-inflammatory M1 marker, top row) and Arg-1 (anti-inflammatory M2 marker, bottom row). Compared to the inflammation control group, H_2_-treated wounds show a marked decrease in iNOS positive cells (brown areas) and a concurrent increase in Arg-1 expression, indicative of a phenotypic switch towards tissue-repairing M2 macrophages. Scale bars: 100 μm.


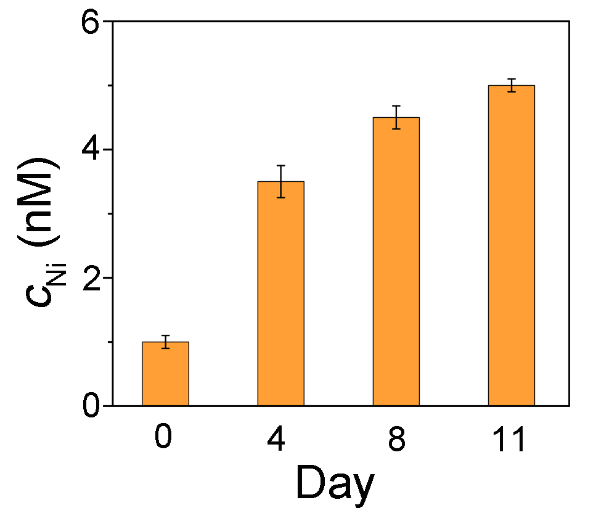


**Fig. S47** The Pd-Ni_5_P_4_/DCEFS system underwent an 11-day treatment period, with ICP-MS testing performed following three daily treatment sessions.


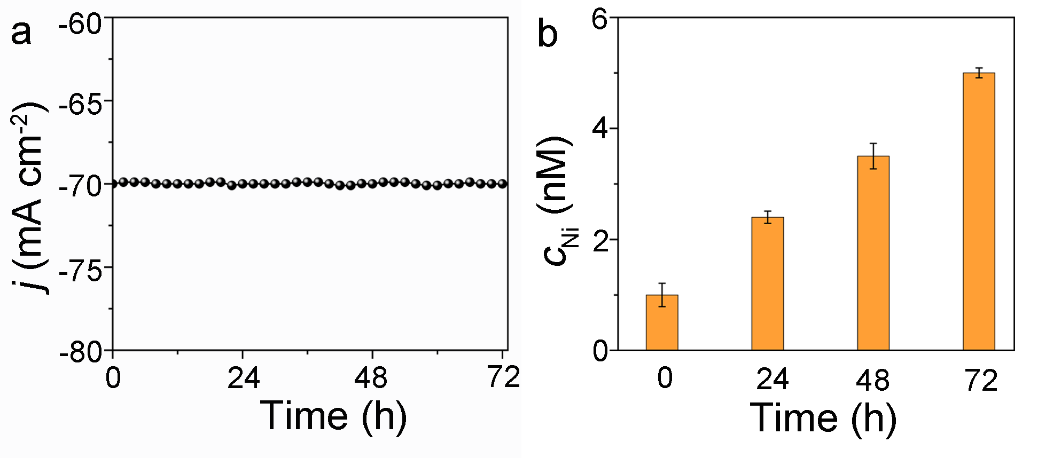


**Fig. S48 a** Stability test conducted at -0.25 V vs. RHE for a duration of 72.0 hours. **b** ICP-MS analysis of the electrolyte sampled at regular intervals during the testing period.


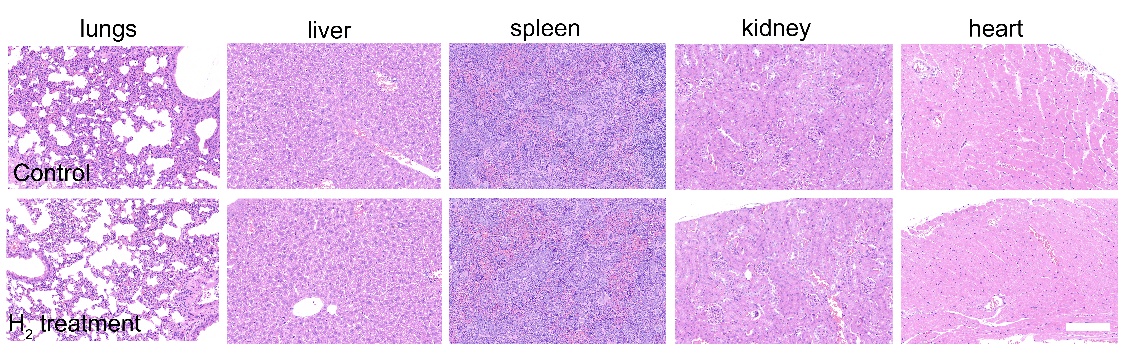


**Fig. S49** Histopathological assessment of major organs in diabetic mice following H_2_ treatment. Representative hematoxylin and eosin (H&E) stained tissue sections from the lungs, liver, spleen, kidney, and heart of diabetic mice in the control group (top row) and the H_2_ treatment group (bottom row). Histological analysis revealed no significant pathological alterations in the organs of H_2_-treated mice compared to the control group, indicating the favorable systemic biocompatibility and safety of the hydrogen therapy. Scale bars: 200 μm.


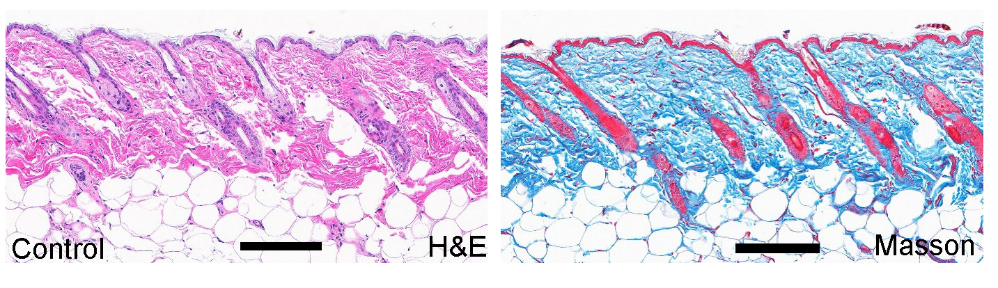


**Fig. S50** Hematoxylin-eosin (H&E) staining (top panel) and Masson’s trichrome staining of normal skin in diabetic mice under an applied potential of -0.25 V. Scale bars: 100 μm.

**Table S1** Summary of the chemical valence states and XPS fitting parameters for the surface elements of Pd-Ni_5_P_4_

| Peak Assignment | Binding Energy (eV) | FWHM (eV) | Area (%) | References |
| --- | --- | --- | --- | --- |
| Ni-P (Ni^δ+^) | 853.3 | 1.89 | 39.0 | Adv. Funct. Mater. 2025, 35, 2412685; Appl. Catal. B-Environ. 2023, 324, 122207. |
| Ni-O (Ni^2+^) | 856.6 | 2.47 | 39.0 |  |
| Ni-O (Ni^3+^) | 859.9 | 2.11 | 22.0 |  |
| Pd-P (Pd^δ+^) | 337.3 | 0.95 | 82.3 | Angew. Chem. Int. Ed. 2025, 64, e202515805; ACS Catal. 2022, 12, 15193–15206. |
| Pd-O (Pd^2+^) | 338.8 | 1.09 | 17.7 |  |
| M-P (P 2p 3/2) | 129.0 | 1.22 | 30.3 | Adv. Funct. Mater. 2025, 35, 2412685; Appl. Catal. B-Environ. 2023, 324, 122207. |
| M-P (P 2p 1/2) | 130.0 | 1.34 | 19.1 |  |
| P-O | 133.8 | 1.35 | 50.6 |  |

**Table S2** Comparison sensors performance of Pd-Ni_5_P_4_/DCEFS with other electrochemical NO Sensors

| electrode design | E | media | LOD | References |
| --- | --- | --- | --- | --- |
| Sb_2_O_4_ | 0.84 V | pH 7.4 | 3.98 nM | J. Colloid. Interface. Sci. 581, 465-474 (2024) |
| PLLA–PTMC | 0.80 V | pH 7.4 | 3.97 nM | Nat. Commun. 11, 3207 (2020) |
| Ni-N_4_ | 0.83 V | pH 7.4 | 1.80 nM | Nat. Commun. 11, 3188 (2020) |
| SWNTs@PEDOT/PDMS | 0.80 V | pH 7.4 | 1.6 nM | Small 16(9), e1903204 (2020) |
| N-G/FePc/Nafion/PLL ITO | 0.90 V | pH=7.4 | 180.0 nM | Anal. Chem. 90(7), 4438-4444 (2018) |
| Au/PDMS | 0.85 V | / | 1.0 nM | Anal. Chem. 90(12), 7158-7163 (2018) |
| Au NTs/TiO_2_ NWs/Au NTs/PDMS | 0.80 V | 5-HT solution | 16.0 nM | Anal. Chem. 90(10), 5977-5981 (2018) |
| CNT-AuNT-PDMS | 0.80 V | / | 0.80 nM | Angew. Chem. Int. Ed. Engl. 56(32), 9454-9458 (2017) |
| FGPC/AuNPs | 0.78 V |  | 3.2 nM | Sci. Rep. 7, 6446 (2017) |
| （TTBA-rGO)/ZnO | -0.95 V | pH=7.0 | 7.7 nM | Small 13(26), 1700502 (2017) |
| TiO_2_/CdS | 0.85 V | pH=4.0 | 3.4 nM | J. Electroanal. Chem. 781, 371-376 (2016) |
| Au NTs/PDMS | 0.85 V | pH=7.4 | 3.0 nM | Angew. Chem. Int. Ed. Engl. 55(14), 4537-4541 (2016) |
| PFNGS | -0.60 V | pH=7.4 | 1.0 nM | J. Mater. Chem. B. 4(27), 4780-4789 (2016) |
| Au NPs-3DGH | 0.81 V | pH=7.4 | 9.0 nM | ACS Appl Mater Inter. 7(4), 2726-2734 |

**Table S3** Comparison of the HER performance of Pd-Ni_5_P_4_/DCEF with reported highly active electrocatalysts under neutral conditions

| Catalyst | Electrolyte | Overpotential (mV) | References |
| --- | --- | --- | --- |
| B-CoP/CNT | PBS (pH=7.0) | -79.0 | Angew. Chem. Int. Ed., 59(10), 4154 – 4160 (2020) |
| CoP/Co-MOF | PBS (pH=7.0) | -49.0 | Angew. Chem. Int. Ed., 14(131), 4679–4684 (2019) |
| N−Ni | PBS (pH=7.0) | -64.0 | J. Am. Chem. Soc., 139(35),  12283−12290 |
| CrOx/Cu–Ni | PBS (pH=7.0) | -48.0 | Nat. energy 4, 107–114 (2019) |
| RuSex–RuNC | 1.0 M Kpi 2.0 (pH=7.0) | -29.0 | Nat. Commun., 13, 6260 (2022) |
| Ru-WO3−x/CP | PBS (pH=7.0) | -86.0 | Nat. Commun., 13, 5382 (2022) |
| Ni/NiMoN | PBS pH = 6.8 | -37.0 | Nano Energy, 78,105375 (2020) |
| Ru/Nb2O5 | PBS (pH=7.0) | -31.0 | Energy Environ. Sci., 17(14), 5091–5101 (2022) |
| Ni0.1Co0.9P | PBS (pH=7.0) | -125.0 | Angew. Chem. Int. Ed.,130(47), 15445 –15449 (2024) |
| SiO2/PPy NTs–CFs | PBS (pH=7.0) | -70.0 | Angew. Chem. Int. Ed., 56(28), 8120 –8124 (2017) |
| Ni–Mo(2.5) | PBS (pH=7.0) | -37.0 | Adv. Sci., 11(39), 2403752 (2024) |
| Ru/Mo2CTx | PBS (pH=7.0) | -73.0 | Adv. Funct. Mater., 33(16), 2214375 (2023) |
| CoP/Ti | PBS (pH=7.0) | -78.0 | Angew. Chem. Int. Ed., 137(15), e202422091 (2025) |
| Pd-Ni_5_P_4_ | PBS (pH=7.4) | -85.0 | This work |

**Table S4** Hematological analysis report of diabetic mice after treatment

| Full Name | Abbreviation | Results | Unit |
| --- | --- | --- | --- |
| White Blood Cell count | WBC | 6.6 | 10^9^/L |
| Lymphocyte Count | Lymph# | 4.6 | 10^9^/L |
| Monocyte Count | Mon# | 0.2 | 10^9^/L |
| Granulocyte Count | Gran# | 1.8 | 10^9^/L |
| Lymphocyte Percentage | Lymph% | 69.3 | % |
| Monocyte Percentage | Mon% | 2.7 | % |
| Granulocyte Percentage | Gran% | 28.0 | % |
| Red Blood Cell Count | RBC | 9.53 | 10^12^/L |
| Hemoglobin | HGB | 146 | g/L |
| Hematocrit | HCT | 53.7 | % |
| Mean Corpuscular Volume | MCV | 56.4 | fL |
| Mean Corpuscular Hemoglobin | MCH | 15.3 | pg |
| Mean Corpuscular Hemoglobin Concentration | MCHC | 271 | g/L |
| Red Cell Distribution Width | RDW | 13.3 | % |
| Platelet Count | PLT | 1150 | 10^9^/L |
| Mean Platelet Volume | MPV | 5.9 | fL |
| Platelet Distribution Width | PDW | 16.1 |  |
| Plateletcrit | PCT | 0.459 | % |

**Table S5** Blood biochemical analysis report of diabetic mice after treatment

| Full Name | Abbreviation | Results | Unit |
| --- | --- | --- | --- |
| Alanine Aminotransferase | ALT | 85.582 | U/L |
| Aspartate Aminotransferase | AST | 256.872 | U/L |
| Total Bilirubin | TBIL | 84.21 | μmol/L |
| Direct Bilirubin | DBIL | 26.942 | μmol/L |
| Albumin | ALB | 34.826 | g/L |
| Alkaline Phosphatase | ALP | 224.758 | U/L |
| γ-Glutamyl Transferase | γ-GT | 1.296 | U/L |
| Total Bile Acid | TBA | 4.986 | μmol/l |
| Urea | UREA | 9.436 | mg/dL |
| Creatinine | CREA | 24.424 | μmol/L |
| Uric Acid | UA | 319.956 | μmol/L |

**Table S6** Hematological analysis report of diabetic mice without treatment

| Full Name | Abbreviation | Results | Unit |
| --- | --- | --- | --- |
| White Blood Cell count | WBC | 6.4 | 10^9^/L |
| Lymphocyte Count | Lymph# | 4.4 | 10^9^/L |
| Monocyte Count | Mon# | 0.2 | 10^9^/L |
| Granulocyte Count | Gran# | 1.8 | 10^9^/L |
| Lymphocyte Percentage | Lymph% | 69.0 | % |
| Monocyte Percentage | Mon% | 3.1 | % |
| Granulocyte Percentage | Gran% | 27.9 | % |
| Red Blood Cell Count | RBC | 7.55 | 10^12^/L |
| Hemoglobin | HGB | 116 | g/L |
| Hematocrit | HCT | 39.7 | % |
| Mean Corpuscular Volume | MCV | 52.7 | fL |
| Mean Corpuscular Hemoglobin | MCH | 15.3 | pg |
| Mean Corpuscular Hemoglobin Concentration | MCHC | 292 | g/L |
| Red Cell Distribution Width | RDW | 14.2 | % |
| Platelet Count | PLT | 765 | 10^9^/L |
| Mean Platelet Volume | MPV | 6.0 | fL |
| Platelet Distribution Width | PDW | 16.6 |  |
| Plateletcrit | PCT | 0.309 | % |

**Table S7** Blood biochemical analysis report of diabetic mice without treatment

| Full Name | Abbreviation | Results | Unit |
| --- | --- | --- | --- |
| Alanine Aminotransferase | ALT | 80.434 | U/L |
| Aspartate Aminotransferase | AST | 200.234 | U/L |
| Total Bilirubin | TBIL | 29.645 | μmol/L |
| Direct Bilirubin | DBIL | 12.305 | μmol/L |
| Albumin | ALB | 21.29 | g/L |
| Alkaline Phosphatase | ALP | 90.259 | U/L |
| γ-Glutamyl Transferase | γ-GT | 0.710 | U/L |
| Total Bile Acid | TBA | 4.737 | μmol/l |
| Urea | UREA | 9.405 | mg/dL |
| Creatinine | CREA | 18.187 | μmol/L |
| Uric Acid | UA | 206.754 | μmol/L |
